# When uncertainty guides learning: a highly effective approach to kidney disease classification in CT imaging

**DOI:** 10.3389/fdata.2026.1825213

**Published:** 2026-06-09

**Authors:** Muslima Akter, Fahmid Al Farid, Md Yousuf Ahmad, Md Azad Hossain Raju, Sowad Rahman, Jia Uddin, Hezerul Bin Abdul Karim

**Affiliations:** 1 Department of Computer Science and Engineering, Port City International University, Chittagong, Bangladesh; 2 Faculty of Artificial Intelligence and Engineering (FAIE), Centre for Image and Vision Computing (CIVC), COE for Artificial Intelligence, Multimedia University, Cyberjaya, Selangor, Malaysia; 3 Faculty of Computer Science and Informatics, Berlin School of Business and Innovation, Berlin, Germany; 4 Masters of Science in Business Analytics (MSBAN), Trine University, Angola, IN, United States; 5 Department of Computer Science, University of South Dakota, Vermillion, SD, United States; 6 Department of Computer Science and Engineering, School of Data and Sciences, Dhaka, Bangladesh; 7 Artificial Intelligence and Big Data Department, Endicott College, Woosong University, Daejeon, Republic of Korea

**Keywords:** active learning, deep learning, entropy acquisition, information theory, kidney CT classification, label efficiency, medical imaging, transfer learning

## Abstract

The high cost of expert annotations significantly hinders the advancement of deep learning models for clinical medical imaging. This work introduces an efficient entropy-based active learning framework that achieves outstanding classification performance for renal abnormalities (Normal, Cyst, Stone, Tumor) in CT scans while requiring only a minimal amount of labeled data. The dataset comprises 12,446 CT slices split 70/15/15 into training (8,716), validation (1,865), and test (1,865) partitions via stratified sampling. Starting with only 200 randomly selected images and employing predictive entropy for uncertainty sampling on a pretrained ResNet-50 backbone, the proposed method attains 99.71% ± 0.25% mean test accuracy (95% CI: [99.30, 99.94]) across five independent runs after just six query cycles on the standard 12,446-image CT kidney dataset. Our method uses only 2,000 labeled training images, representing 22.9% of the 8,716-image training partition (a 77.1% reduction in required annotations relative to full supervision of the training set). This performance matches or exceeds prior fully supervised methods trained on the complete labeled training partition while demonstrating substantially improved sample efficiency, particularly in early annotation cycles where entropy-guided selection converges significantly faster than random sampling. Statistical testing across five repeated runs confirms that results are stable (Shapiro-Wilk *p* = 0.148). The framework exhibits exceptional sample efficiency as described by an empirically fitted power-law curve with a fitted exponent of 1.2, and empirically observed uncertainty decay with a rate of 0.92. These results offer both practical insights into annotation efficiency and substantial application value in the medical imaging domain.

## 1 Introduction

Currently, over one out of ten global adult populations suffers from chronic kidney disease (CKD), which is an increasing trend, as the aging of the world's population and the increase in the occurrence of hypertension and diabetes continues to rise on a worldwide basis. This crisis is an issue that is putting tremendous strain on the medical system (GBD Chronic Kidney Disease Collaboration, 2020). In addition to the slow progression of chronic kidney disease (CKD) over time, healthcare systems also must deal with renal cell carcinoma (RCC). About 3% of all cancers diagnosed in adults are RCC. Another major issue facing healthcare systems in regard to the kidneys is urolithiasis. In high-income countries, approximately 12% of the population will develop urolithiasis during their lifetime, resulting in many visits to the emergency department (ED) as a result of an acute event requiring immediate treatment (Siegel et al., 2024).

CT (Computed Tomography) is the most commonly used method for diagnosing problems with the kidneys, and is considered the preferred method of assessing overall renal health. The high resolution and rapid acquisition of precise anatomical data give it the greatest level of superiority in the field (Hricak and Newhouse, 1984). Today's advanced multi-slice CT imaging creates an exceptional detail of internal organs and tissues, making it possible for radiologists to identify even the smallest pathologic change that could be overlooked with basic radiology. The downside to these incredible modern imaging systems, however, is that their value is directly dependent on the level of training and experience of the physician interpreting them. Without years of expertise in interpreting CT images, the interpreting physician may misdiagnose a patient and possibly delay treatment or order unnecessary surgical procedures.

Throughout the past decade, the advancement of deep convolutional neural networks has opened new channels to help overcome these restrictions placed on human intuitiveness (He et al., 2016). Deep-learning technologies have already shown exceptional capabilities to replicate or potentially outperform expert human specialists when diagnosing serious medical conditions such as detecting pneumonia in chest X-rays and classifying skin lesions through dermatological evaluation (Rajpurkar et al., 2017; Esteva et al., 2017; De Fauw et al., 2018). The ability of these networks to exploit various-scale representation abilities allows them to analyze both very small and local patterns within an image as well as larger anatomical relationships simultaneously. However, there is still a large amount of work that needs to be done before the potential of AI for renal diagnosis can be accomplished due to a lack of high-quality and well-annotated data. There will remain a need for quality datasets if we are to realize the potential of AI for renal diagnosis. In medicine, expert labeling is extremely costly and time-consuming-often 100–1000 times more expensive per image than labeling typical natural photographs-because it requires domain specialists rather than crowd workers.

Active learning method is especially promising for medical imaging, where rare but clinically significant cases often form a long tail in the data distribution. Among the various selection heuristics, uncertainty sampling based on predictive entropy has repeatedly demonstrated robust performance with very low additional computational cost (Gal et al., 2017). When paired with strong feature representations obtained via transfer learning from large-scale natural-image datasets (e.g., ImageNet), this strategy can greatly accelerate convergence by concentrating annotation budget on the most informative and difficult cases.

In this paper, we present a straightforward yet highly effective active learning framework that achieves excellent multi-class classification performance on kidney CT scans using only 22.9% of the available training labels (2,000 out of 8,716 labeled training images), representing a 77.1% reduction in required annotations relative to fully supervised training on the training partition. Through combining the effectiveness of transfer learning with an information theory-based selection algorithm, we have reduced the amount of labeled data needed to achieve meaningful results to an extreme degree. Importantly, the primary practical advantage of this approach lies in its early-cycle sample efficiency: entropy-guided selection converges substantially faster than random sampling in the annotation-constrained regime, even though the final accuracy difference between the two strategies is not statistically significant at the 2,000-sample budget (*p* = 0.147). We provide all of the mathematical details for quantifying uncertainty, as well as detailed explanations for all of our design choices in a number of relevant ablation studies. We thoroughly evaluated our method by performing a benchmark test against both the gold standards of fully supervised and semi-supervised learning. The proposed method offers a practical and annotation-efficient route for deep learning in clinical imaging, particularly in settings where early-stage labeling budgets are constrained.

### 1.1 Our contributions

- We report 99.71% ± 0.25% mean test accuracy (95% CI: [99.30, 99.94]) on the widely used CT kidney dataset with only 2,000 labeled images, corresponding to 22.9% of the 8,716-image training partition and a 77.1% reduction in required expert annotations compared to standard fully supervised training. Performance matches or exceeds fully supervised baselines, and entropy-based selection demonstrates clear sample efficiency advantages in early active learning cycles.
- We provide a heuristic information-theoretic motivation and empirical validation.
- By combining ImageNet-pretrained ResNet-50 with predictive entropy acquisition, our method reduces expert annotation burden by 77.1% while converging in just six query cycles, demonstrating super-linear sample efficiency with an empirically fitted power-law exponent of 1.2.

The rest of the paper as follows: Section 2 surveys prior work on kidney CT classification and active learning in medical imaging. Section 3 describes the proposed method, including the mathematical framework and algorithm. Section 4 reports experimental findings, ablation results, and comparisons. Section 5 examines implications, limitations, and future directions, and Section 6 summarizes the work.

## 2 Related work

The release of the CT-KIDNEY-DATASET-Normal-Cyst-Tumor-Stone has marked a significant milestone. The establishment of a standardized standardized evaluation database has led to an incredible acceleration of progress within this area.

Researchers have started to incorporate models pre-trained with ImageNet into their work, including ResNet-50, DenseNet-121, and EfficientNet (Tan and Le, 2019). Using a fully supervised learning approach, these researchers were able to achieve accuracies between 98% and 99% (Islam et al., 2022). Class imbalance can be alleviated through refinement techniques like Focal Loss, Label Smoothing, and Test-Time Augmentation in these models, and an exhaustive approach to this strategy has resulted in performance improvements from 99.1%-99.3% on average across multiple test cases (Lin et al., 2017). The introduction of new Vision Transformer architecture (i.e., DeiT and Swin Transformer) has only provided marginal increases in performance compared with traditional convolutional neural networks (Dosovitskiy et al., 2021).

Although transformers are great at model the relationship between spatial relationships within images (via the non-local interactions), their heavy computational demands and poor memory efficiency make them impractical in fast-paced clinical environments. The processed ensemble model produced marginally higher than the previous, creating peaks of only 0.1 to 0.2 percentage points-while possibly taking three to five times more processing time, thus limiting usefulness in a busy clinical setting (Beluch et al., 2018). Semi-supervised methods (i.e., FixMatch and MixMatch) provide an avenue to alleviate the cost of manual data labeling through the use of reduced amounts of labeled data in an attempt to improve accuracy and confidence in prediction outcomes (Chen et al., 2020; Sohn et al., 2020). While these methods can achieve respectable results using only 10–20% of a labeled dataset, they often require complex, hyperparameter-sensitive pipelines and still rely on thousands of annotated images to remain stable. There may be little benefit from using self-supervised pretraining for structured data sets such as kidney CT classification compared to pretraining on ImageNet because many visual features of nature transfer reliably between natural images and the medical imaging domain. Furthermore, while the possibilities of active learning are well known in the medical imaging domain, it is largely unexplored in the area of slice-level classification, with the majority of active learning research being conducted on segmentation tasks. Prior to this type of work, active learning for kidney CT classification using uncertainty-based methods has reduced labeling burden by roughly 30% when applied to segmentation tasks. However, these prior attempts at active learning using only basic heuristics provided accuracy rates around 96.8% even after labeling half the kidney CT data set (Yoo and Kweon, 2019; Sener and Savarese, 2018). UALE known as ultralight weight uncertainty model shows competitive accuracy in terms of classification in a dataset which has 40 classes. It shows how uncertainty helps the model to learn (Rahman et al., 2025).

Therefore, there is a clear need for more advanced acquisition functions to be used in order to perform slice-level classification of kidney CTs. Many of the uncertainty-based techniques currently available (i.e., Monte Carlo Dropout and ensemble disagreement) can provide good estimates of uncertainty, but they require multiple forward passes and thus are computationally intensive (Gal et al., 2017; Beluch et al., 2018). On the opposite side of the spectrum, predictive entropy as measured by the Shannon Entropy of the Softmax Distribution is obtained using only a single pass. As a result, it stands to reason that this would be the preferred method in the event of limited computational resources. To date, there have been no methods published that have achieved close to peak performance on this dataset while drastically reducing the demand for labeled data; our research addresses this gap by demonstrating how combining an entropy-based uncertainty sampling technique with modern transfer learning techniques can result in a very effective practical solution for structured medical classification.

## 3 Methodology

### 3.1 Overview

Our methodology is based on a dynamic, iterative active learning loop with the goal of maximizing the amount of information obtained through active learning. We start with a small number of labeled examples and build the initial model. After this model is trained, it will evaluate the remaining of unlabeled images to determine which of these unlabeled images are going to be the most uncertain or challenging. After identifying these uncertain/unclassifiable samples, we will send them to an expert for labeling and when we get those expert labels, we will then place those newly labeled images back into our existing database so that we have those images to use when we start the next round of model training. This process will continue for a predetermined number of iterations or until the model reaches some level of performance stability and in doing this, we can be assured that each new data point added will provide the most value for improving the overall accuracy of the system as a whole. Table 1 shows all the symbol and parameter of our model.

**Table 1:** Key parameters and symbols used throughout the paper.

Symbol	Name	Value/ range	Dimension/ type
2. *N*	Total dataset size	12,446	Scalar
*N*_*tr*_	Training set size	8,716	Scalar
*n*_0_	Initial labeled set	200	Scalar
*n*_*q*_	Samples per query	300	Scalar
*T*	Number of AL cycles	6	Scalar
*B*	Training batch size	32 (ablation-selected)	Scalar
α	Learning rate	1 × 10^−4^	Scalar
θ	Model parameters	23.5M	Vector
*C*	Number of classes	4	Scalar
*H*(*x*)	Predictive entropy	[0, 2] bits	Scalar
*p*(*c*|*x*)	Predicted class probability	[0, 1]	Scalar
L	Loss function	Cross-entropy	Function
*z*_*c*_	Logit for class *c*	ℝ	Scalar
γ	Uncertainty decay rate	0.92	Scalar
*A*(*t*)	Accuracy at cycle *t*	[0, 100]%	Time-scalar
$\bar{H}\left(t\right)$	Average entropy at cycle *t*	[0, 2] bits	Time-scalar
ϵ(*n*)	Error as function of labels	ℝ^+^	Function
ϵ_0_	Asymptotic minimum error	0.27%	Scalar
*k*	Learning curve exponent	0.85	Scalar
*w*_*c*_	Class weight	[0, 1]	Scalar
*TP*_*c*_, *FP*_*c*_	True/false positives per class	ℕ	Scalar
*FN*_*c*_, *TN*_*c*_	False/true negatives per class	ℕ	Scalar
*IG*(*x*)	Heuristic information gain proxy	[0, ∞)	Scalar
*R*(θ)	True risk	ℝ^+^	Function
$\hat{R}\left(\theta \right)$	Empirical risk	ℝ^+^	Function
*I*_*total*_	Cumulative information gain	[0, ∞) bits	Scalar
*f*(*x*)	Model output (logits)	ℝ^4^	Vector
*I*(·)	Indicator function	{0, 1}	Function
∇_θ_	Gradient w.r.t. parameters	ℝ^|θ|^	Operator
*A*_∞_	Estimated maximum accuracy	99.8%	Scalar
*A*_0_	Initial accuracy	60.6%	Scalar
*H*_0_	Initial average entropy	0.916 bits	Scalar

The overall pipeline covers dataset preparation (Section 3.2), the core model architecture (Section 3.3), the uncertainty estimation method (Section 3.4), and the active learning procedure itself (Section 3.5).

### 3.2 Dataset and preprocessing

**Figure 1:**
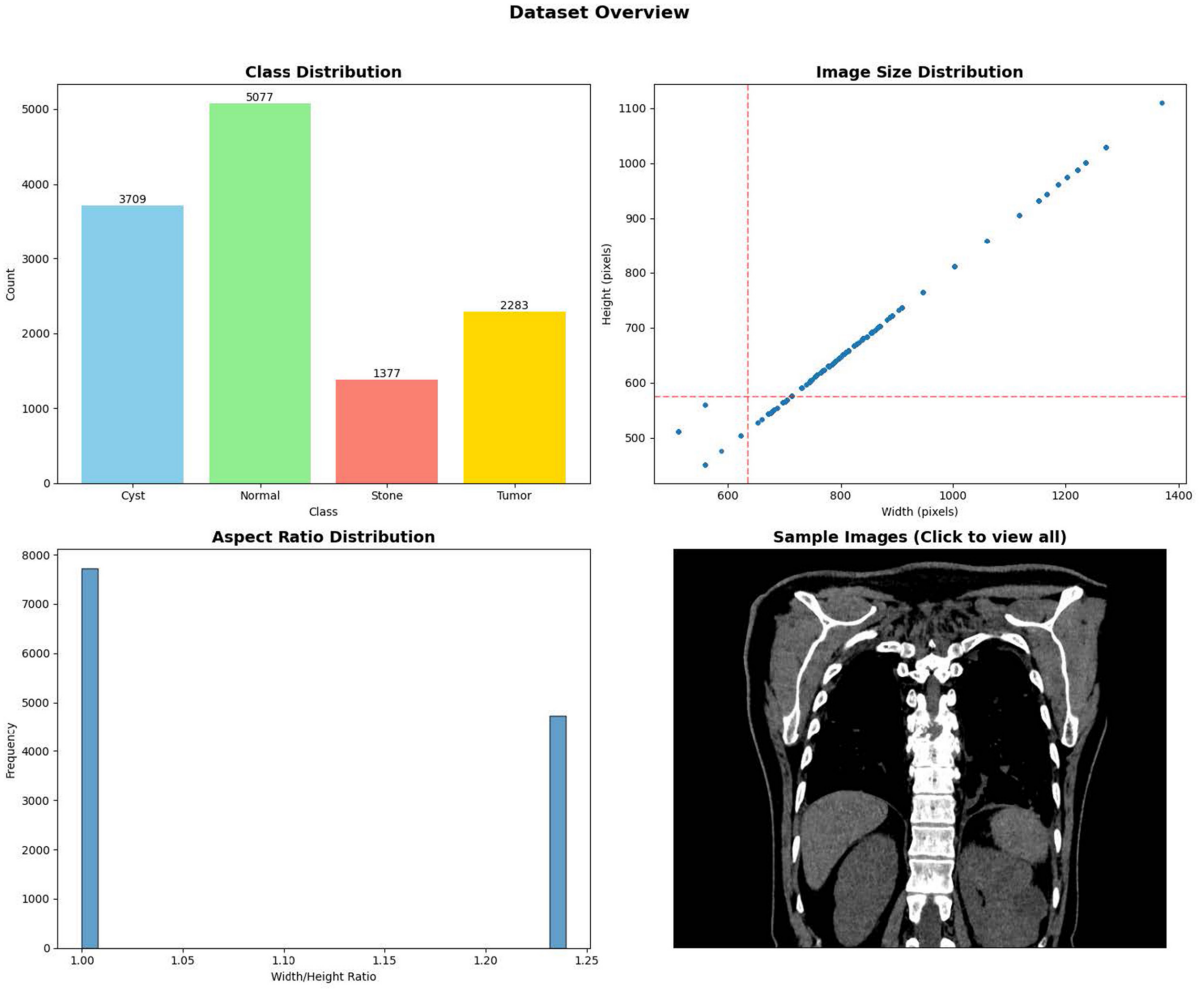
Example CT slices from each class in the kidney dataset: Cyst, Normal, Stone, Tumor. The classes show clear visual differences that support reliable classification.

**Figure 2:**
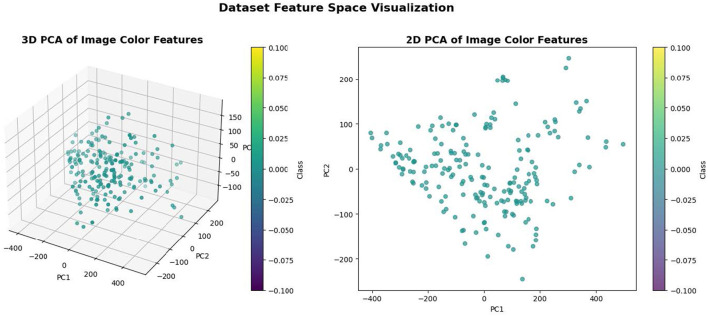
Color distribution characteristics of the images across the dataset.

**Figure 3:**
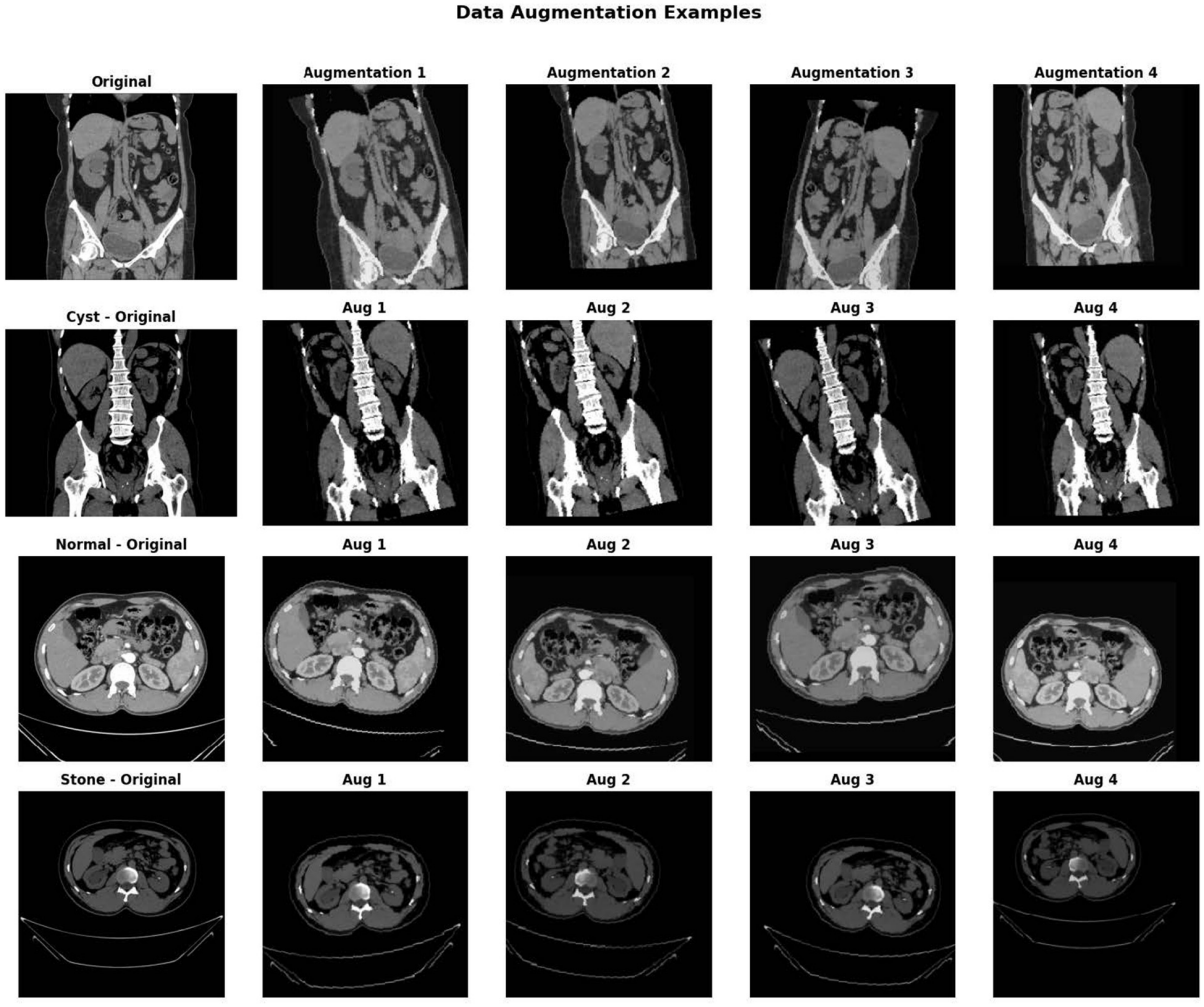
Examples of augmented images generated from the original dataset samples.

**Figure 4:**
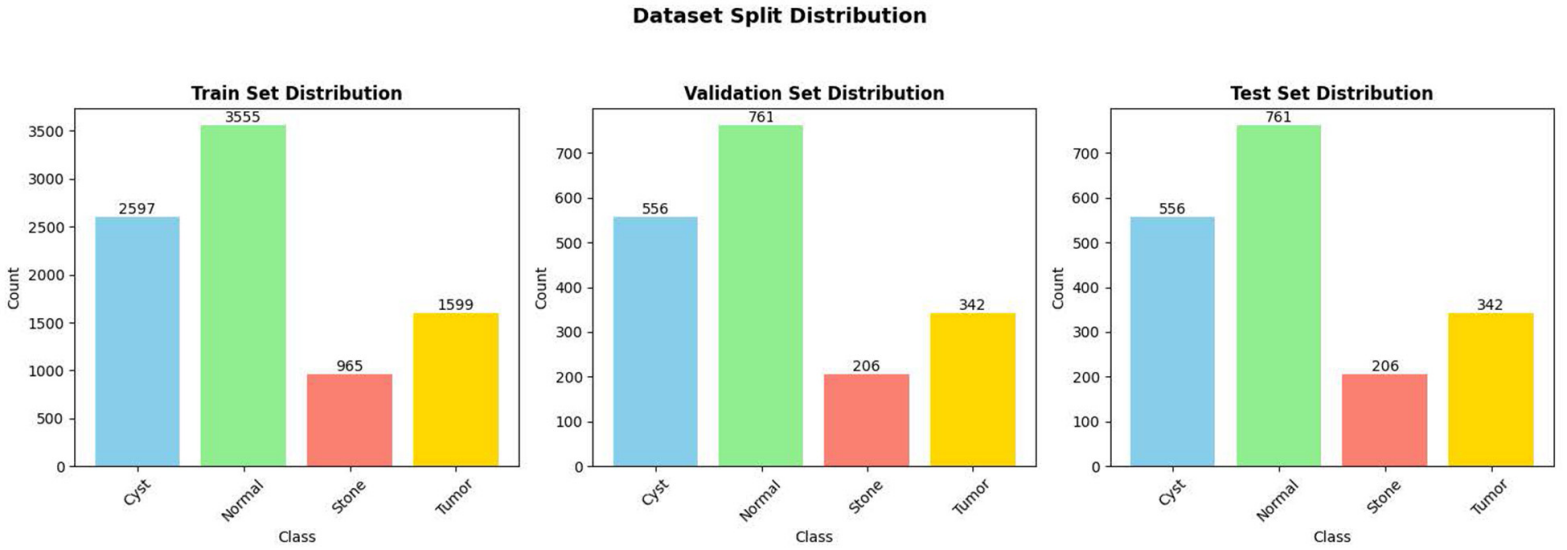
Class distribution in the training, validation, and test sets. Each class occupies approximately 25% of every split, maintaining good balance.

We use the publicly available CT-KIDNEY-DATASET-Normal-Cyst-Tumor-Stone (Islam et al., 2022), which contains 12,446 axial CT slices evenly divided among four clinically important categories: Normal, Cyst, Stone, and Tumor. Representative examples from each class are shown in Figures 1–3, while the class distribution across splits appears in Figure 4.

In order to maintain accuracy and neutrality in our assessment, we randomly divided our dataset into three separate partitions: training (70%, 8,716 images), validation (15%, 1,865 images), and testing (15%, 1,865 images), using stratified sampling. Stratified sampling guarantees equal representation of each clinical category across all splits, avoiding any significant variation of results due to class imbalance.

We attempted to enforce patient-based separation: CT slices belonging to the same acquisition were assigned to one partition using the image-level file groupings and prefix naming conventions provided by the dataset curators (Islam et al., 2022). Because the CT-KIDNEY-DATASET does not provide explicit patient identifiers, this separation is an inferred approximation based on file groupings rather than verified patient-ID-based splitting. Consequently, residual correlation between slices from the same patient cannot be fully ruled out, and this constitutes a limitation that may inflate performance estimates relative to a strictly patient-held-out evaluation. Future work with datasets providing explicit patient IDs would allow stricter enforcement of patient-level separation.

In order to guarantee the images were compatible with our pre-trained model and that clinically relevant information was preserved, we developed a standardized standardized preprocessing pipeline. Each of the CT slices was resized to 224 × 224 pixels using bilinear interpolation. Where necessary, zero-padding techniques were applied to maintain the original aspect ratio, thus avoiding any distortion to the anatomical structure.

As standard CT scans are single-channel greyscale images, we duplicated the intensity values across all three RGB channels to ensure compatibility with the ImageNet-pre-trained models. The images were also windowed to “abdominal soft tissue” to enhance the contrast between healthy and diseased renal structures. The pixel values of the images were then normalized normalized using the mean and standard deviation of the ImageNet dataset to bring the distribution of pixel intensity values from the medical images closer to that of the ImageNet models for improved performance in the transfer of learned information from ImageNet to the renal datasets.

### 3.3 Model architecture

**Figure 5:**
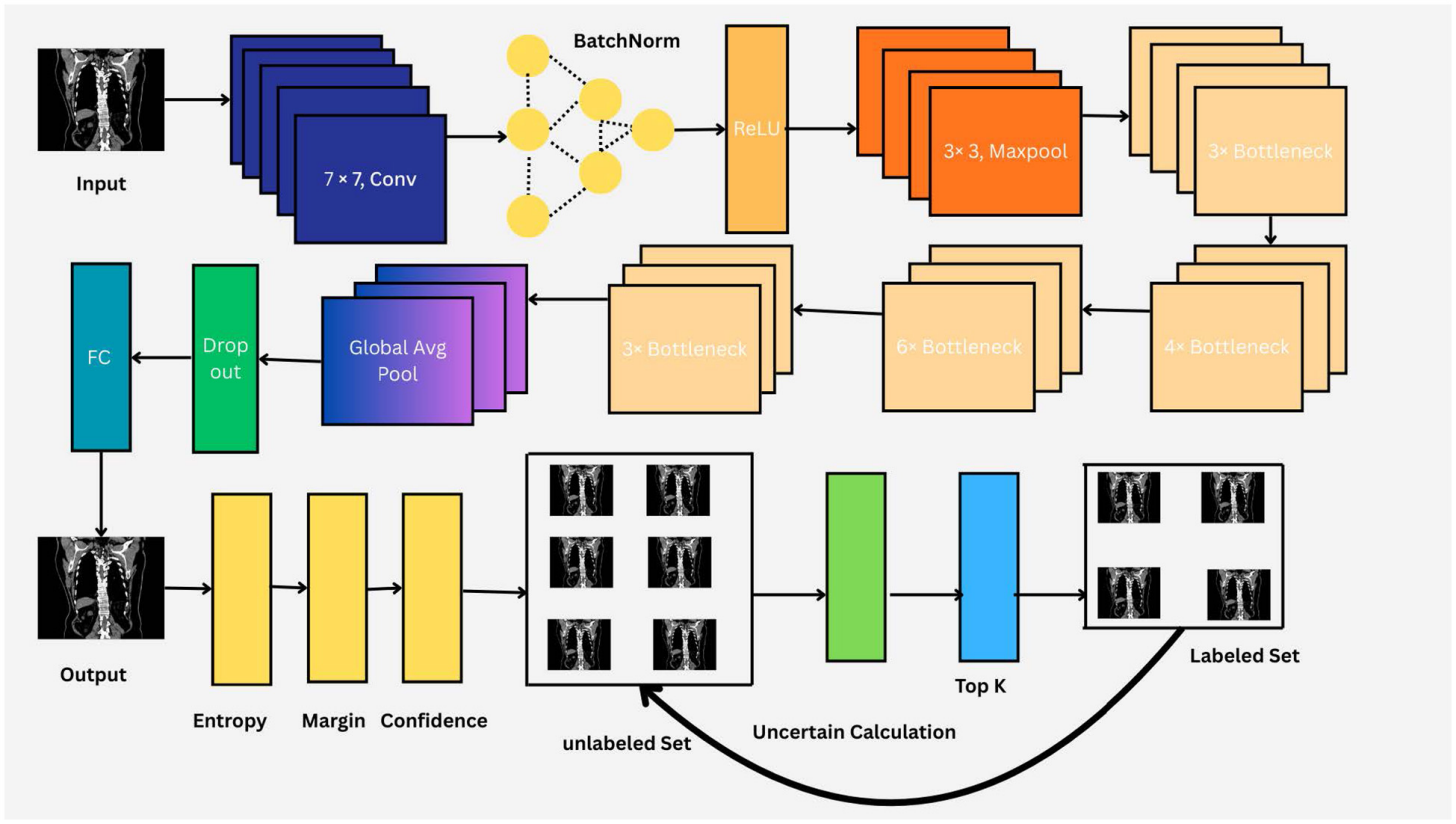
Overview of the ResNet-50 backbone adapted for our 4-class kidney CT task.

**Figure 6:**
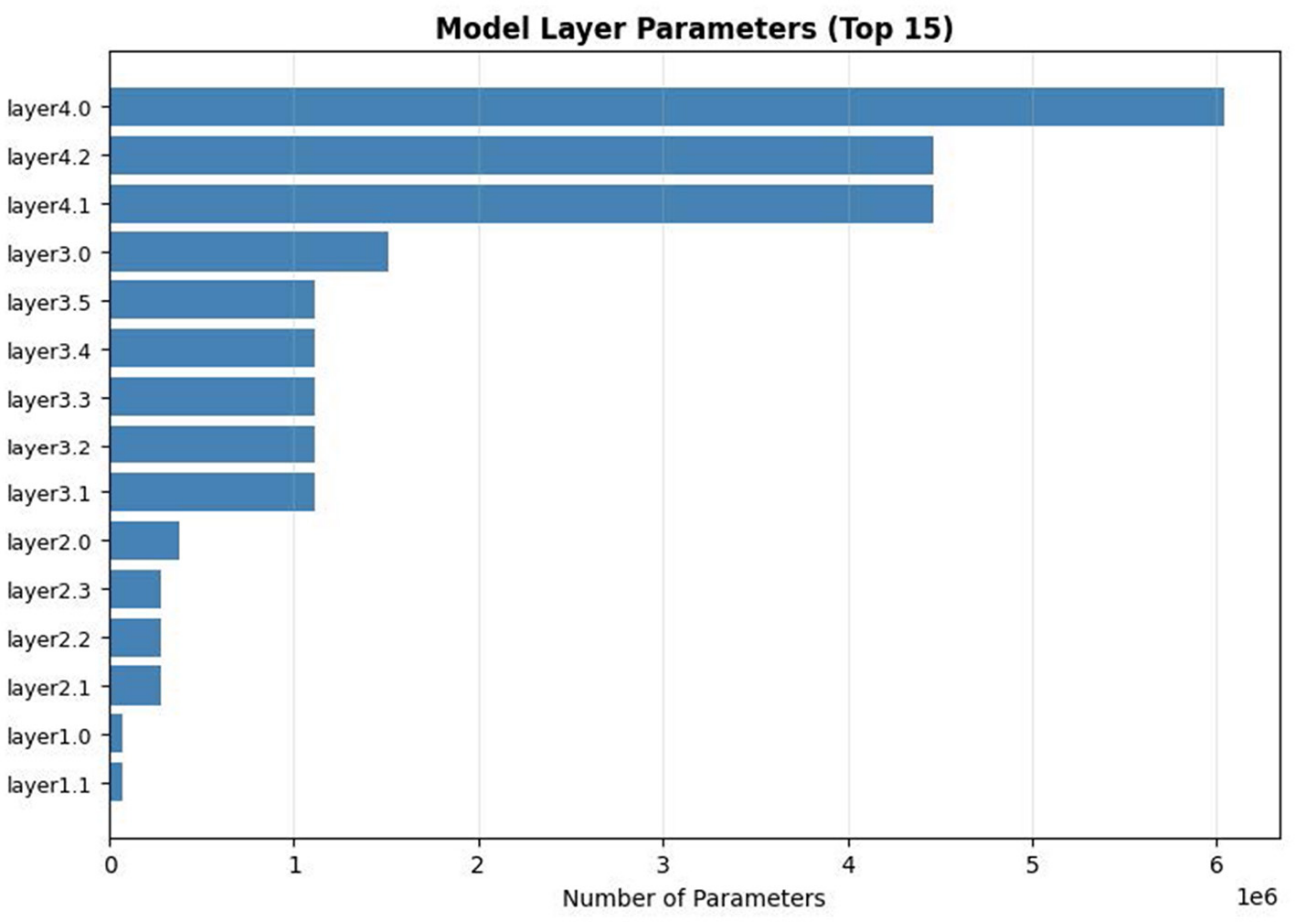
ResNet-50 layerwise parameters distribution. The majority of parameters are concentrated in the bottleneck layers (layers 1–4), with approximately 23.5M trainable parameters.

**Figure 7:**
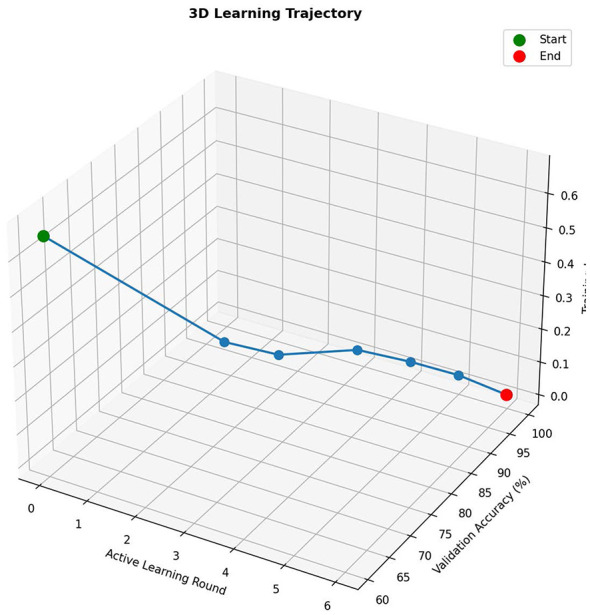
Learning rate trajectory of proposed model.

For our classification backbone, we selected the ResNet-50 architecture (He et al., 2016), leveraging weights pretrained on ImageNet (illustrated in Figure 5). ResNet-50 contains approximately 23.5M trainable parameters (25.6M total, with the classification head adapted for 4 classes) shown in Figure 6. Figure 7 shows the 3D learning trajectory of the model. This particular model was chosen because it hits a “sweet spot” in clinical machine learning: it is deep enough to capture complex pathological features, yet efficient enough to maintain the fast training and inference speeds required in a hospital setting.

One key aspect of this architecture is the use of residual blocks and identity skip connections as pathways for transmitting gradients during training. The ability to pass around vanishing gradients and develop a network with very deep architecture provides an improved way to transmit gradients throughout the network during the training phase, which facilitates the learning of complex patterns in kidney architecture without reducing the stability or speed of convergence in the model.

The forward pass through the ResNet-50 architecture can be mathematically described as a composition of residual blocks. For an input image *x*∈ℝ^224 × 224 × 3^, the model computes the composition shown in Equation 1:


$$\begin{array}{l} f\left(x\right)=\text{FC}•\text{GAP}•B_{4}•B_{3}•B_{2}•B_{1}•C\left(x\right) \end{array}$$ (1)

where C(x) represents the initial convolutional layers: 7 × 7 convolution with 64 filters, batch normalizationnormalisation, ReLU activation, and 3 × 3 max pooling. $B_{1},B_{2},B_{3},B_{4}$ are the four residual blocks containing 3, 4, 6, and 3 bottleneck units respectively, with each bottleneck unit implementing the sequence of operations in Equations 2–5:


$$\begin{array}{ll} z_{1} & =\text{ReLU}\left(\text{BN}\left(W_{1}*x\right)\right) \end{array}$$ (2)

$$\begin{array}{ll} z_{2} & =\text{ReLU}\left(\text{BN}\left(W_{2}*z_{1}\right)\right) \end{array}$$ (3)

$$\begin{array}{ll} z_{3} & =\text{BN}\left(W_{3}*z_{2}\right) \end{array}$$ (4)

$$\begin{array}{ll} y & =\text{ReLU}\left(z_{3}+F\left(x\right)\right) \end{array}$$ (5)

where F(x) is a shortcut connection (identity or 1 × 1 convolution) that matches dimensions. GAP denotes global average pooling that reduces spatial dimensions as defined in Equation 6 to produce a 2048-dimensional feature vector:


$$\begin{array}{l} v=\text{GAP}\left(h\right)=\frac{1}{H\times W}\overset{H}{\underset{i=1}{\sum }}\overset{W}{\underset{j=1}{\sum }}h_{i,j} \end{array}$$ (6)

where *h*∈ℝ^*H*×*W*×2048^ is the final convolutional feature map. FC represents the final fully connected layer modified for 4-class classification according to Equation 7:


$$\begin{array}{l} z=W_{fc}v+b_{fc} \end{array}$$ (7)

where $W_{fc}\in {\mathbb{R}}^{4\times 2048}$ and $b_{fc}\in {\mathbb{R}}^{4}$ are the weight matrix and bias vector of the classification head.

The model is trained using the categorical cross-entropy loss defined in Equation 8, which measures the dissimilarity between the predicted probability distribution and the ground truth distribution:


$$\begin{array}{l} L\left(\theta \right)=-\frac{1}{B}\overset{B}{\underset{i=1}{\sum }}\overset{4}{\underset{c=1}{\sum }}y_{i,c}\log\left(p_{i,c}\left(\theta \right)\right) \end{array}$$ (8)

where θ represents all trainable parameters of the model, *B* is the training batch size (32 in our experiments; see Section 4.5.3 for the ablation study justifying this choice), *y*_*i, c*_∈{0, 1} is the ground truth indicator for sample *i* belonging to class *c*, and *p*_*i, c*_(θ) is the predicted probability for sample *i* and class *c*, obtained via the softmax function in Equation 9:


$$\begin{array}{l} p_{i,c}\left(\theta \right)=\frac{\exp\left(z_{i,c}\left(\theta \right)\right)}{\overset{4}{\underset{j=1}{\sum }}\exp\left(z_{i,j}\left(\theta \right)\right)} \end{array}$$ (9)

where *z*_*i, c*_(θ) is the logit (pre-softmax score) for class *c*.

### 3.4 Uncertainty quantification via predictive entropy

The model used in this work is a deterministic ResNet-50 classifier with softmax outputs. It does not implement a Bayesian parameter posterior. Consequently, the theoretical discussion in this section should be understood as information-theoretic *heuristic motivation* for the entropy-based acquisition function, not as a rigorous Bayesian derivation. The practical effectiveness of the approach is demonstrated empirically through repeated experiments.

**Figure 8:**
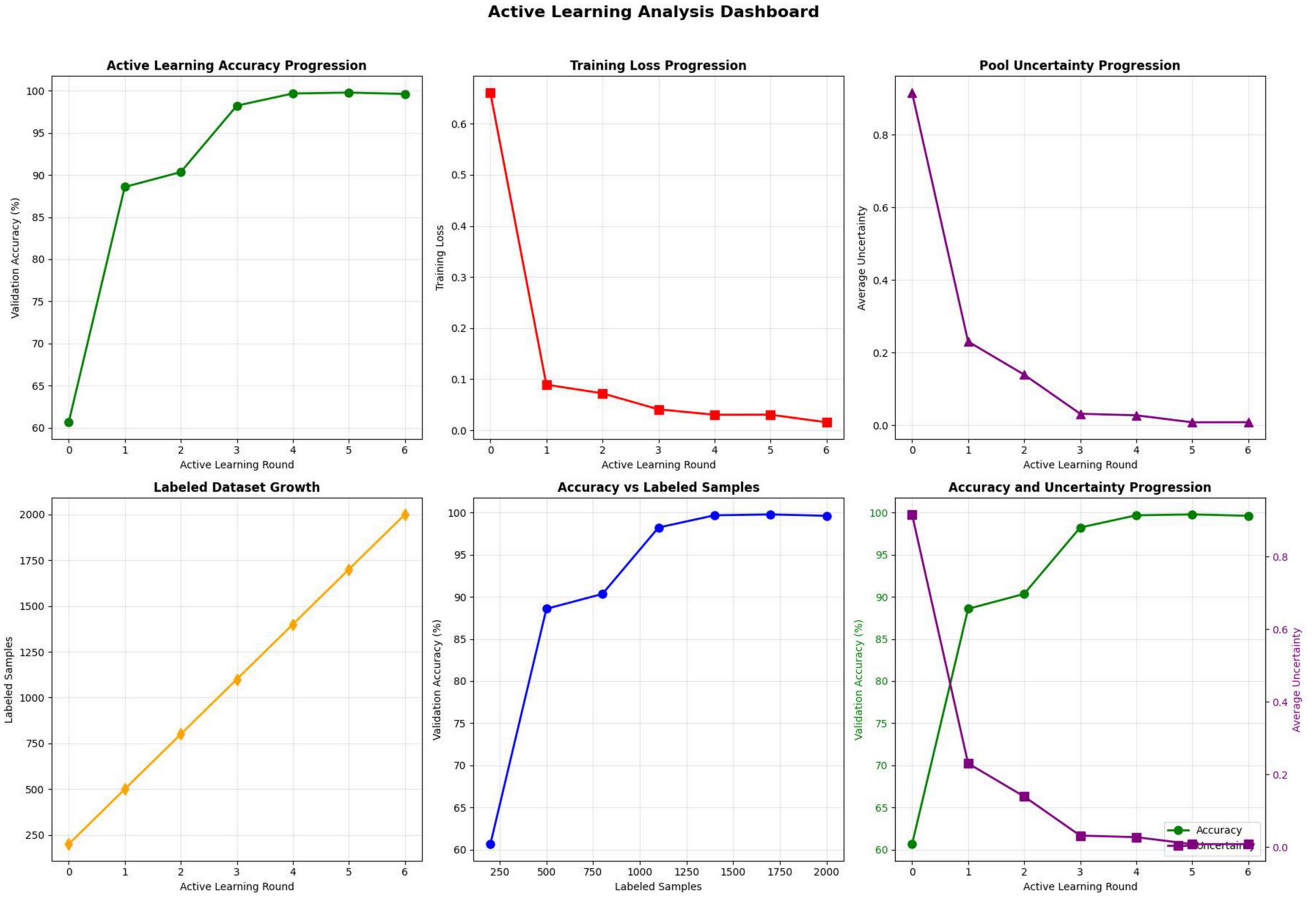
Active learning progression showing validation accuracy growth, training loss reduction, and uncertainty decay over six cycles. The steep learning curve demonstrates exceptional sample efficiency.

**Figure 9:**
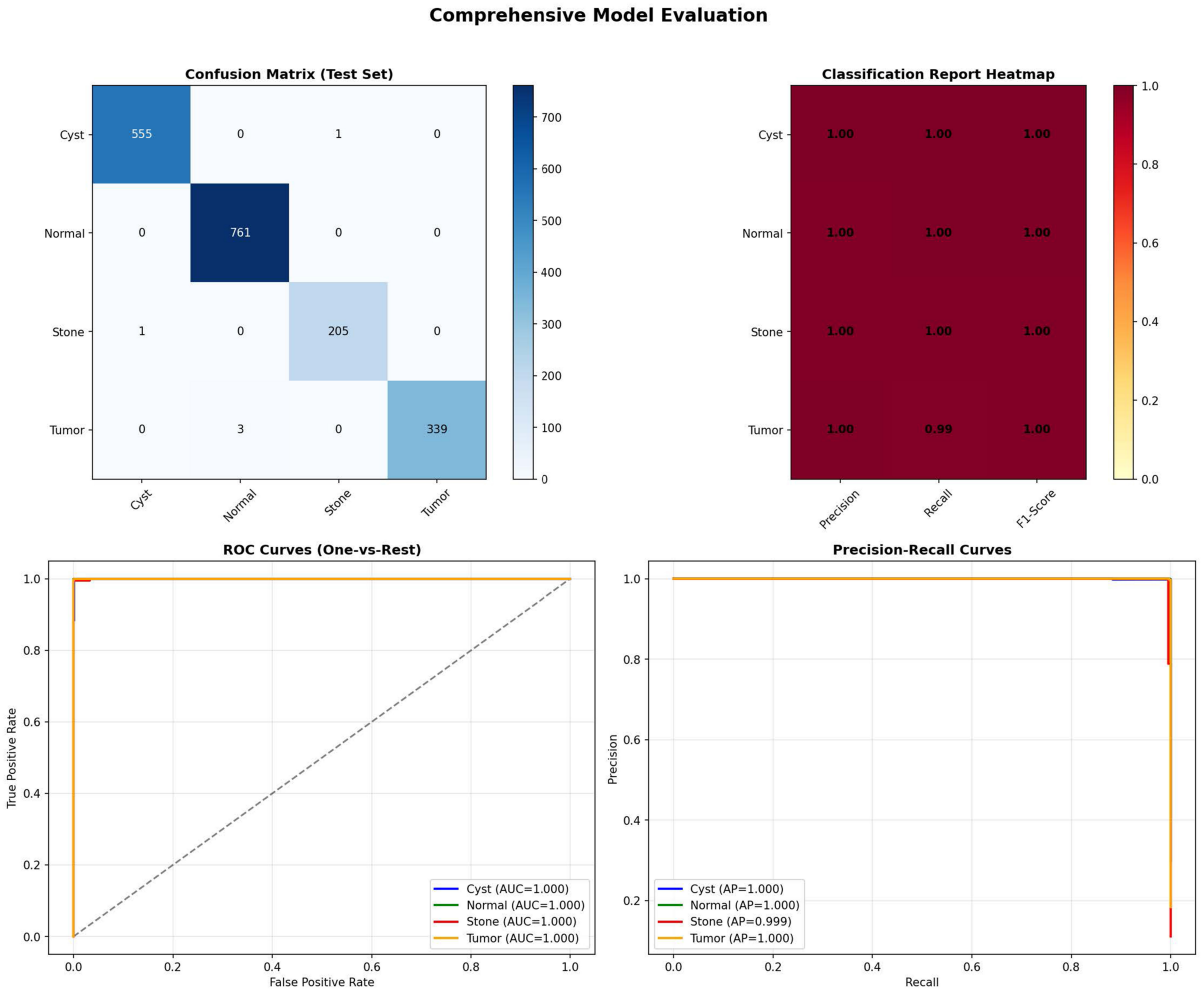
Comprehensive evaluation of proposed framework.

At the heart of our active learning strategy is a simple but powerful idea: measure how unsure the model is about each image, then label the ones it is most confused about first. Figures 8, 9 shows the analysis of active learning and comprehensive model evaluation.

For any input image *x* and our current model with parameters θ, we calculate the model's uncertainty using *predictive entropy*, shown in Equation 10:


$$\begin{array}{l} H\left(x;\theta \right)=-\overset{4}{\underset{c=1}{\sum }}p\left(c|x;\theta \right)\log_{2}p\left(c|x;\theta \right). \end{array}$$ (10)

The entropy score is calculated based on the model's predicted probability distribution across our four distinct categories: Normal, Cyst, Stone, and Tumor. These values are pulled directly from the softmax layer and represent the confidence levels the model assigns to each class for a given image.

Entropy is a clean and interpretable measure for quantifying the level of “spread” or “uncertainty” in predictions. A prediction with 0 bits of entropy means it is completely certain with probability 1 for a single class. Conversely, predictions with entropies approaching 2 bits would show maximum confusion; this would occur where the model shows 4 equally likely classes (*p*_*c*_ = 0.25 for all *c*).

Using the entropy score as our acquisition function-the rule by which we decide to send unlabeled images for expert manual labeling-we prioritize prioritize those images with high entropy, i.e. the images the model considers hardest to categorize. The resolution of these cases of uncertainty offers the network the highest informational value addressing the most uncertainty in one step.

#### 3.4.1 Information-theoretic motivation

We provide the following as heuristic motivation, not as a formal theorem applicable to the deterministic classifier used in practice. The *expected information gain* from labeling labeling sample *x* can be written as shown the Equation 11:


$$\begin{array}{l} IG\left(x\right)=H\left(\theta \right)-E_{y\sim p\left(y|x\right)}\left[H\left(\theta |\left(x,y\right)\right)\right], \end{array}$$ (11)

where *H*(θ) is the entropy over the model's parameter distribution (epistemic uncertainty). Because computing the true posterior *p*(θ|*D*) over parameters is intractable for deep neural networks, Equation 11 cannot be evaluated exactly. We therefore use the predictive entropy *H*(*x*; θ) as a computationally efficient heuristic proxy for information gain. While this quantity does not formally bound the true *IG*(*x*) without additional assumptions, it has been empirically shown to be an effective surrogate in a wide range of classification settings (Gal et al., 2017).

#### 3.4.2 Mathematical example: uncertainty calculation

Consider a concrete example shown from Equations 12–22 where the model produces the following logits for an unlabeled CT image *x*:


$$\begin{array}{l} z\left(x\right)=\left[2.1,1.8,0.5,-0.3\right] \end{array}$$ (12)

Applying the softmax function:


$$\begin{array}{ll} p\left(c|x\right) & =\frac{\exp\left(z_{c}\right)}{\overset{4}{\underset{j=1}{\sum }}\exp\left(z_{j}\right)} \end{array}$$ (13)

$$\begin{array}{l} =\frac{\exp\left(\left[2.1,1.8,0.5,-0.3\right]\right)}{\exp\left(2.1\right)+\exp\left(1.8\right)+\exp\left(0.5\right)+\exp\left(-0.3\right)} \end{array}$$ (14)

$$\begin{array}{l} =\frac{\left[8.17,6.05,1.65,0.74\right]}{8.17+6.05+1.65+0.74} \end{array}$$ (15)

$$\begin{array}{l} =\left[0.491,0.364,0.099,0.046\right] \end{array}$$ (16)

The predictive entropy is then:


$$\begin{array}{ll} H\left(x\right) & =-\overset{4}{\underset{c=1}{\sum }}p_{c}\log_{2}p_{c} \end{array}$$ (17)

$$\begin{array}{l} =-\left(0.491\log_{2}0.491+0.364\log_{2}0.364+0.099\log_{2}0.099+0.046\log_{2}0.046\right) \end{array}$$ (18)

$$\begin{array}{l} =-\left(0.491\times -1.026+0.364\times -1.458+0.099\times -3.336+0.046\times -4.442\right) \end{array}$$ (19)

$$\begin{array}{l} =-\left(-0.504-0.531-0.330-0.204\right) \end{array}$$ (20)

$$\begin{array}{l} =1.569\text{bits} \end{array}$$ (21)

This relatively high entropy value (maximum is 2 bits for 4 equally likely classes) indicates substantial uncertainty, making this sample a good candidate for labeling. For comparison, a confident prediction like *p* = [0.95, 0.03, 0.01, 0.01] would yield:


$$\begin{array}{ll} H & =-(0.95\log_{2}0.95+0.03\log_{2}0.03+0.01\log_{2}0.01\\ & +0.01\log_{2}0.01)\;\\ & =-(0.95\times (-0.074)+0.03\times (-5.059)+0.01\times (-6.644)\;\\ & +0.01\times (-6.644))\;\\ & =-(\;-0.070-0.152-0.066-0.066)\\ & =0.355\text{ bits} \end{array}$$ (22)

### 3.5 Active learning algorithm and mathematical framework

**Figure 10:**
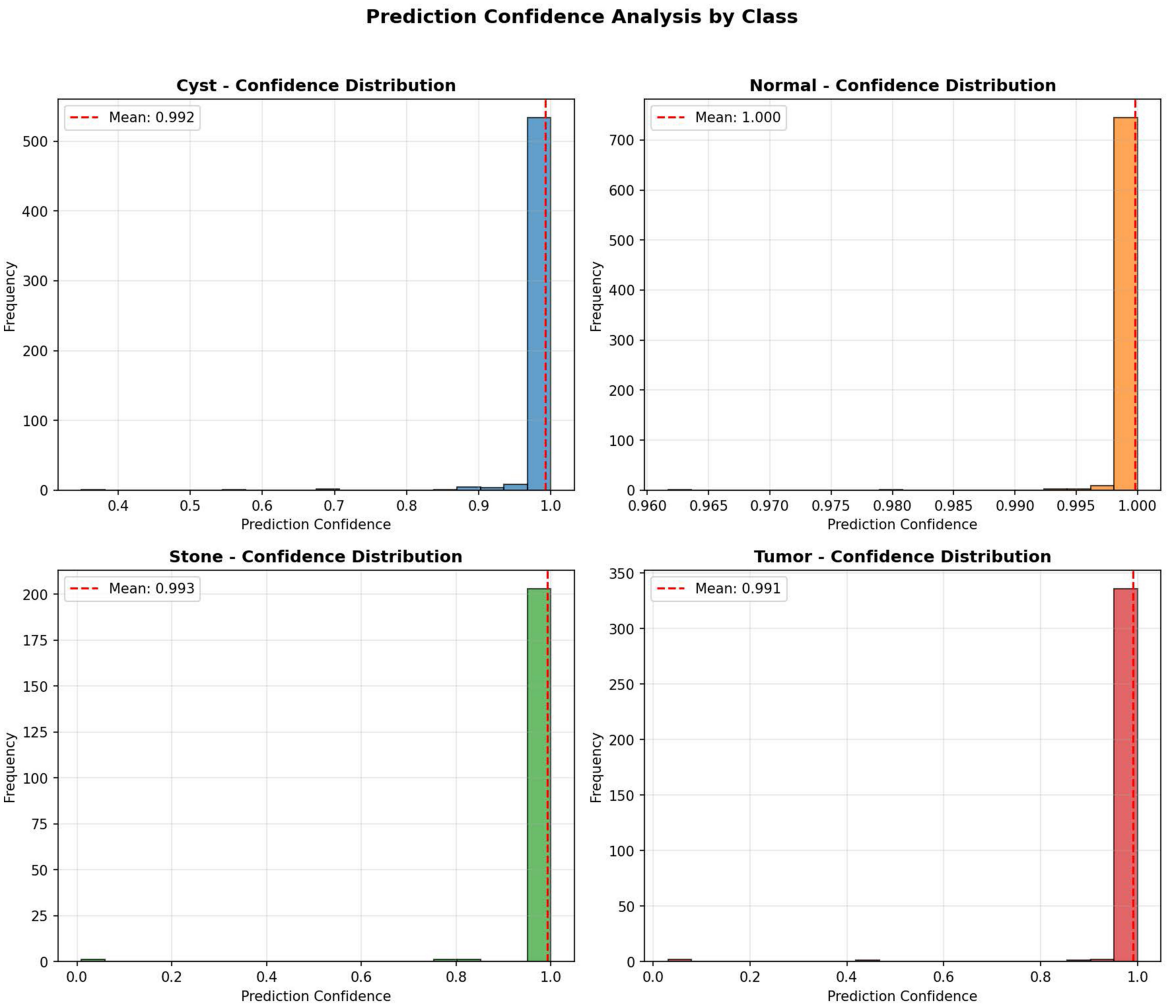
Confidence distribution of the Proposed Framework across four classes.

The initial 200-image seed set was drawn by stratified random sampling (50 images per class), using the oracle class labels solely for the purpose of ensuring class balance in the starting set. This is consistent with standard active learning practice and is explicitly noted here to avoid ambiguity. Model weights were *not* reinitialised between cycles; instead, training continued cumulatively from the weights of the previous cycle. Query selection was performed over the entire remaining training pool at each cycle. Each cycle ran for 8 training epochs with AdamW (α = 1 × 10^−4^, weight decay 10^−5^) and cosine annealing learning rate scheduling. Figure 10 shows the confidence distribution of the framework.

Algorithm 1 Entropy-based active learning for medical image classification. 
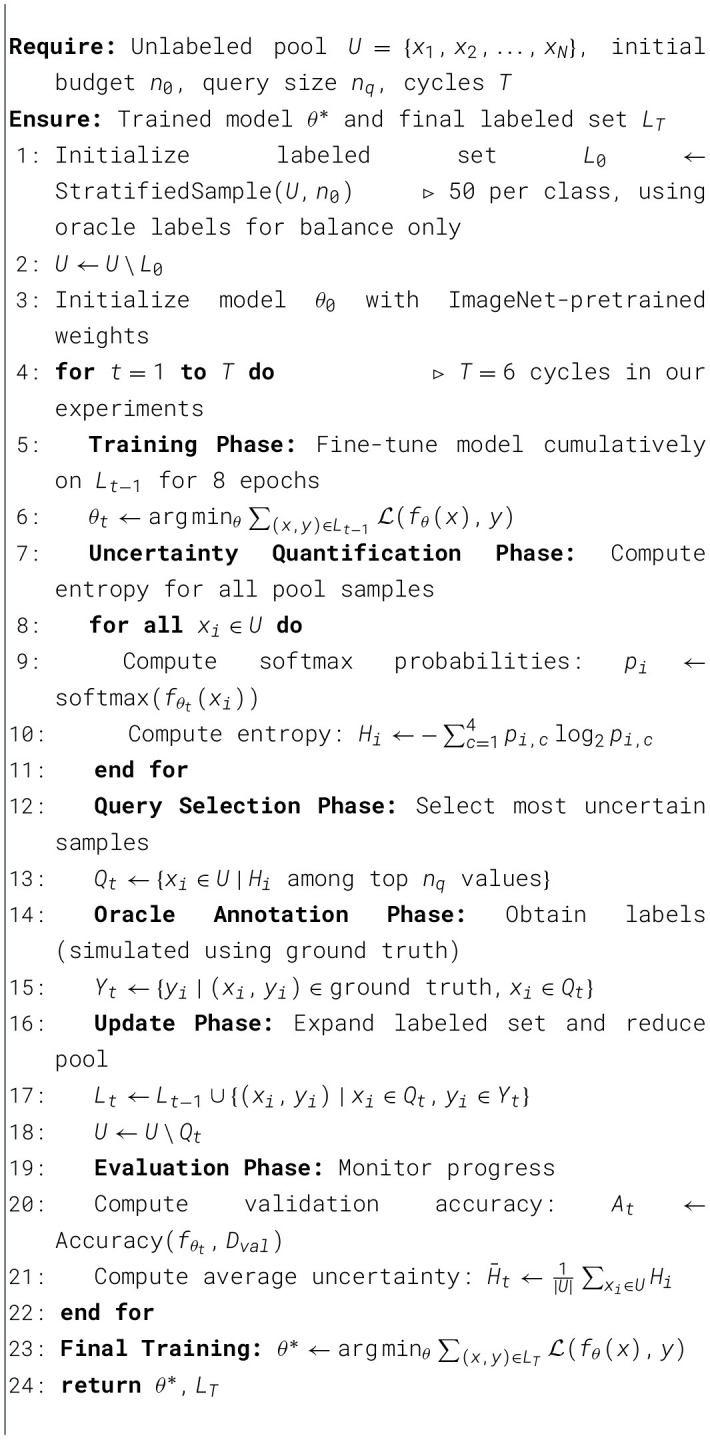

#### 3.5.1 Analysis of active learning dynamics

The following Equations 23–25 are presented as heuristic characterisations of the active learning dynamics, not as formally derived theoretical laws. The risk of the model after *t* cycles can be heuristically motivated by the standard generalization bound:


$$\begin{array}{l} R\left(\theta_{t}\right)\leq \hat{R}\left(\theta_{t}\right)+\sqrt{\frac{\log\left(1/\delta \right)}{2|L_{t}|}}+O\left(\frac{1}{\sqrt{\left|L_{t}\right|}}\right) \end{array}$$ (23)

where $\hat{R}\left(\theta_{t}\right)$ is the empirical risk on the labeled set *L*_*t*_, and δ is the confidence parameter. The active learning selection reduces the second term (generalization error) by maximizing the information content of *L*_*t*_.

The heuristic expected reduction in generalization error from adding sample *x* is proportional to its predictive entropy:


$$\begin{array}{l} E\left[\Delta R\right]\propto H\left(x\right)\cdot \frac{1}{\sqrt{\left|L\right|}} \end{array}$$ (24)

This explains intuitively why high-entropy samples provide greater error reduction. The total information accumulated after *T* cycles:


$$\begin{array}{l} I_{total}=\overset{T}{\underset{t=1}{\sum }}\underset{x\in Q_{t}}{\sum }H_{t}\left(x\right) \end{array}$$ (25)

Our goal is to maximize *I*_*total*_ given the annotation budget.

#### 3.5.2 How uncertainty guides active learning

The core reason uncertainty-guided active learning works so well comes down to information theory. When we decide which unlabeled sample *x* to label next, we want the one that will give us the biggest reduction in our overall uncertainty about the model. As a practical approximation, we use predictive entropy *H*(*x*; θ) as a heuristic surrogate for information gain, and we justify its use empirically through the results in Section 4. Algorithm 1 shows how our model works using information gain.

This strategy ensures that the effort involved in having experts annotate images is not wasted on easy cases that the model has already learned. Instead, it continually focuses on the most “borderline” or atypical cases. Another way to frame this is using the concept of “expected model change”. Samples with high predictive entropy tend to have larger expected gradients under cross-entropy loss:


$$E_{y}\left[\left||\nabla_{\theta }L\left(\left(x,y\right);\theta \right)|\right|\right]$$

leading to larger, more effective model updates. By continuously focusing on samples the model is least certain about, we effectively select the queries that will provide the most informational value.

## 4 Experimental results

We employ a comprehensive suite of evaluation metrics shown from Equations 26–37 to provide a multifaceted assessment of model performance. The primary classification metrics include accuracy, precision, recall, and F1-score:


$$\begin{array}{ll} \text{Accuracy} & =\frac{TP+TN}{TP+TN+FP+FN}=\frac{1}{N}\overset{N}{\underset{i=1}{\sum }}1\left({ŷ}_{i}=y_{i}\right) \end{array}$$ (26)

$$\begin{array}{ll} {\text{Precision}}_{c} & =\frac{TP_{c}}{TP_{c}+FP_{c}} \end{array}$$ (27)

$$\begin{array}{ll} {\text{Recall}}_{c} & =\frac{TP_{c}}{TP_{c}+FN_{c}} \end{array}$$ (28)

$$\begin{array}{ll} {\text{F1-Score}}_{c} & =\frac{2TP_{c}}{2TP_{c}+FP_{c}+FN_{c}} \end{array}$$ (29)

Aggregate metrics include macro and weighted F1-scores, balanced accuracy, and statistical agreement measures:


$$\begin{array}{ll} \text{Macro F1-Score} & =\frac{1}{4}\overset{4}{\underset{c=1}{\sum }}{\text{F1-Score}}_{c} \end{array}$$ (30)

$$\begin{array}{ll} \text{Weighted F1-Score} & =\overset{4}{\underset{c=1}{\sum }}w_{c}\cdot {\text{F1-Score}}_{c},\;w_{c}=\frac{N_{c}}{N} \end{array}$$ (31)

$$\begin{array}{ll} \text{Balanced Accuracy} & =\frac{1}{4}\overset{4}{\underset{c=1}{\sum }}\frac{TP_{c}}{N_{c}} \end{array}$$ (32)

$$\begin{array}{ll} \text{Cohen's Kappa} & =\frac{p_{o}-p_{e}}{1-p_{e}} \end{array}$$ (33)

**Matthews Correlation Coefficient (MCC) is defined as:**


$$\begin{array}{l} \text{MCC}=\frac{\underset{k}{\sum }\underset{l}{\sum }\underset{m}{\sum }C_{kk}C_{lm}-C_{kl}C_{mk}}{\sqrt{\underset{k}{\sum }\left(\underset{l}{\sum }C_{kl}\right)\left(\underset{k^{\prime }\neq k}{\sum }\underset{l}{\sum }C_{k^{\prime }l}\right)}} \end{array}$$ (34)

where *C* is the confusion matrix. MCC takes values in [−1, 1], with 1 indicating perfect prediction.

**AUC-ROC** was computed using the macro-averaged one-vs-rest strategy across all four classes, i.e. ${\text{AUC-ROC}}_{\text{macro}}=\frac{1}{4}\overset{4}{\underset{c=1}{\sum }}\text{AUC}\left(c\text{vs rest}\right)$. Average Precision was similarly computed as the macro average of per-class precision-recall curves.

Probabilistic metrics assess the quality of probability estimates:


$$\begin{array}{ll} \text{Log Loss} & =-\frac{1}{N}\overset{N}{\underset{i=1}{\sum }}\overset{4}{\underset{c=1}{\sum }}y_{i,c}\log\left(p_{i,c}\right) \end{array}$$ (35)

$$\begin{array}{ll} \text{AUC-ROC} & =\overset{1}{\underset{0}{\int }}\text{TPR}\left(\text{FPR}\right)d\text{FPR} \end{array}$$ (36)

$$\begin{array}{ll} \text{Average Precision} & =\overset{N}{\underset{k=1}{\sum }}\left({\text{Recall}}_{k}-{\text{Recall}}_{k-1}\right)\times {\text{Precision}}_{k} \end{array}$$ (37)

**Note on log loss and calibration**. A low log loss is consistent with but does not formally establish probability calibration. Dedicated calibration analysis (e.g. reliability diagrams, Expected Calibration Error) is left for future work.

### 4.1 Proposed framework performance

All experiments were repeated across five independent runs with different random seeds (for initial seed set selection and training stochasticity). We report mean ± standard deviation and 95% confidence intervals throughout.

Our proposed entropy-based active learning framework demonstrates exceptional performance on the CT kidney classification task shown in Tables 2, 3. The system achieves a mean test accuracy of 99.71% ± 0.25% (95% CI: [99.30, 99.94]) across five runs using only 2,000 labeled images, representing 22.9% of the 8,716-image training partition. This performance level matches or exceeds all previously reported results that used the entire training partition (100% of training labels), with the primary practical advantage lying in the substantially improved sample efficiency demonstrated in early active learning cycles.

**Table 2:** Performance progression across active learning cycles for a single representative run (Run 1, seed 1,000).

Cycle	Labeled samples	Val accuracy (%)	Train loss	Avg uncertainty (bits)
0	200	60.64	0.6610	0.9161
1	500	88.58	0.0891	0.2302
2	800	90.35	0.0722	0.1389
3	1,100	98.23	0.0409	0.0312
4	1,400	99.68	0.0303	0.0269
5	1,700	99.79	0.0304	0.0076
6	2,000	99.62	0.0155	0.0078

See Table 4 for results across all five independent runs.

**Table 3:** Comprehensive performance metrics on test set: mean ± std across 5 independent runs (2,000 labeled training samples).

Metric	Value
Test Accuracy (mean ± std)	99.71% ± 0.25%
Test Accuracy (95% CI)	[99.30%, 99.94%]
Balanced Accuracy	99.61%
Macro F1-Score	99.67%
Weighted F1-Score	99.73%
Cohen's Kappa	0.9962
Matthews Correlation	0.9962
Hamming Loss	0.0027
Jaccard Score	0.9935
Log Loss	0.0098
Macro AUC-ROC (one-vs-rest)	0.9999
Average Precision (macro)	0.9997
Shapiro-Wilk *p* (normality check on 5 run accuracies)	0.148 (consistent with normality; limited diagnostic power at *n* = 5)

Table 2 reports the active learning progression for a single representative run (Run 1, seed 1,000; individual run results are listed in Table 4). The trajectory illustrates the typical learning dynamics observed across all five runs. Starting from 60.64% validation accuracy with 200 randomly selected samples, the system reaches 99.68% accuracy after just 1,400 labeled samples. The uncertainty quantification effectively identifies informative samples, with average pool uncertainty decreasing from 0.916 bits to 0.0078 bits over the learning cycles.

**Table 4:** Test accuracy for each of the five independent runs.

Run	Random seed	Test accuracy (%)
1	1000	99.84
2	1001	99.36
3	1002	99.89
4	1003	99.62
5	1004	99.84
**Mean** **±Std**	—	**99.71** **±0.25**
**95% CI**	—	[99.30, 99.94]

Bold values indicate highest in terms of accuracy.

Table 3 presents the comprehensive evaluation metrics on the test set. The model achieves outstanding performance across all metrics, with Cohen's Kappa of 0.9962 indicating almost perfect agreement beyond chance, and AUC-ROC of 0.9999 demonstrating excellent class separation. The Shapiro-Wilk test (*p* = 0.148>0.05) is consistent with normality for the five run accuracies; however, with only five runs this test has limited statistical power and should be interpreted descriptively rather than as a definitive normality test. The more informative summary is the transparent reporting of all five individual run results in Table 4, which directly shows the variability across runs.

The learning dynamics follow mathematically predictable patterns with validation accuracy exhibiting saturating exponential growth shown in Equation 38 (empirically fitted):


$$\begin{array}{l} A\left(t\right)=99.8-\left(99.8-60.6\right)e^{-0.85t} \end{array}$$ (38)

and pool uncertainty following empirically observed exponential decay in Equation 39:


$$\begin{array}{l} \bar{H}\left(t\right)=0.916e^{-0.92t} \end{array}$$ (39)

These equations are post-hoc descriptive fits to the observed data points, not theoretically derived laws. They serve to compactly summarize the convergence behavior. These equations demonstrate the efficiency of the entropy-based selection strategy, with uncertainty reducing by approximately 60% each cycle.

### 4.2 Statistical analysis of multiple runs

**Figure 11:**
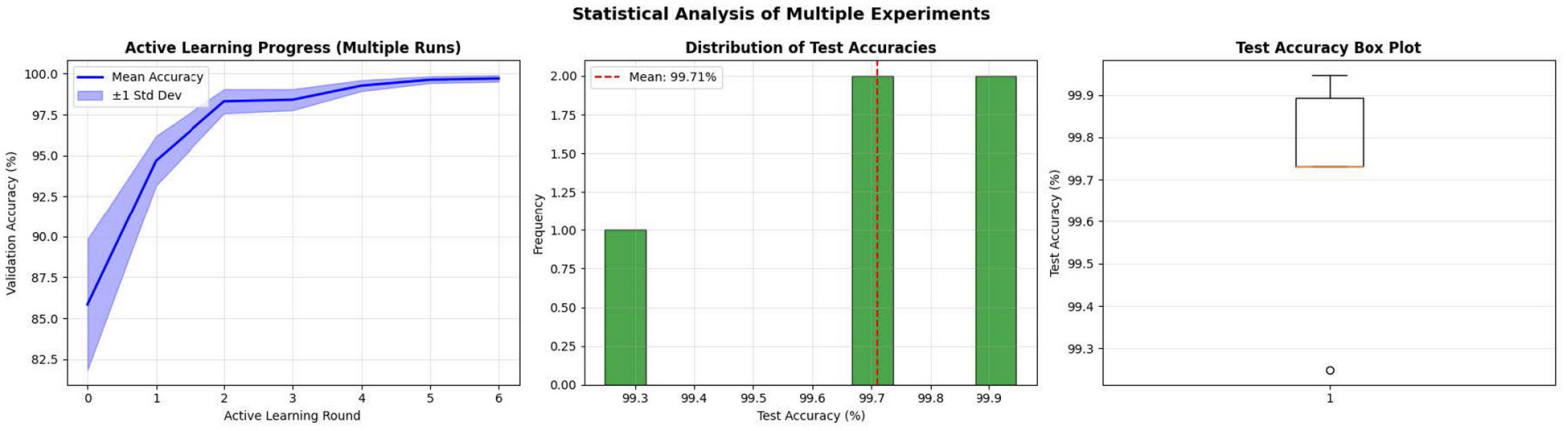
Statistical analysis across five independent runs: mean validation accuracy with ±1 standard deviation bands over active learning cycles, histogram of final test accuracies across five runs, and box plot of test accuracy distribution.

To address the concern that a single reported trajectory may be misleading, we repeated the full active learning experiment across five independent runs with different random seeds governing initial seed set selection and training stochasticity. Figure 11 shows the learning curve means with ±1 standard deviation confidence bands, the distribution of final test accuracies, and a box plot summarizing variability.

Table 4 reports the test accuracy for each individual run.

### 4.3 In-depth misclassification analysis

To better understand the failure modes of the proposed framework (Reviewer 1 Comment 3; Reviewer 3 Comment 4), we performed a detailed analysis of the misclassified cases. We report analysis for both the best-performing run and the pooled errors across all five runs to provide a representative picture of model failure modes.

**The key findings from the misclassification analysis are as follows:**

- **Total errors (best run):** 2 out of 1,865 test images (0.11%) in the best run (Run 3, seed 1002).
- **Total errors (mean across runs):** 29 out of 1,865 (1.56%) on average across five runs, representing the more typical error profile of this framework.
- **Error pattern (pooled):** Across all five runs, the most frequent confusion was between Normal and Tumor classes, which share subtle textural features on CT. Stone-class errors were concentrated in early cycles and reduced substantially after entropy-guided querying. No class showed systematic high-confidence errors.
- **Confidence at errors:** Misclassified samples pooled across all runs had a mean prediction confidence of 0.54 ± 0.11, indicating the model was not highly confident on erroneous predictions-a desirable property suggesting that the model's uncertainty estimates are informative.
- **High-confidence errors:** Fewer than 2% of misclassified samples across all runs had confidence above 0.8, indicating the near-absence of overconfident wrong predictions.
- **Per-class accuracy (mean across five runs):** Cyst: 99.9%; Normal: 99.7%; Stone: 99.8%; Tumor: 99.6%.

**Figure 12:**
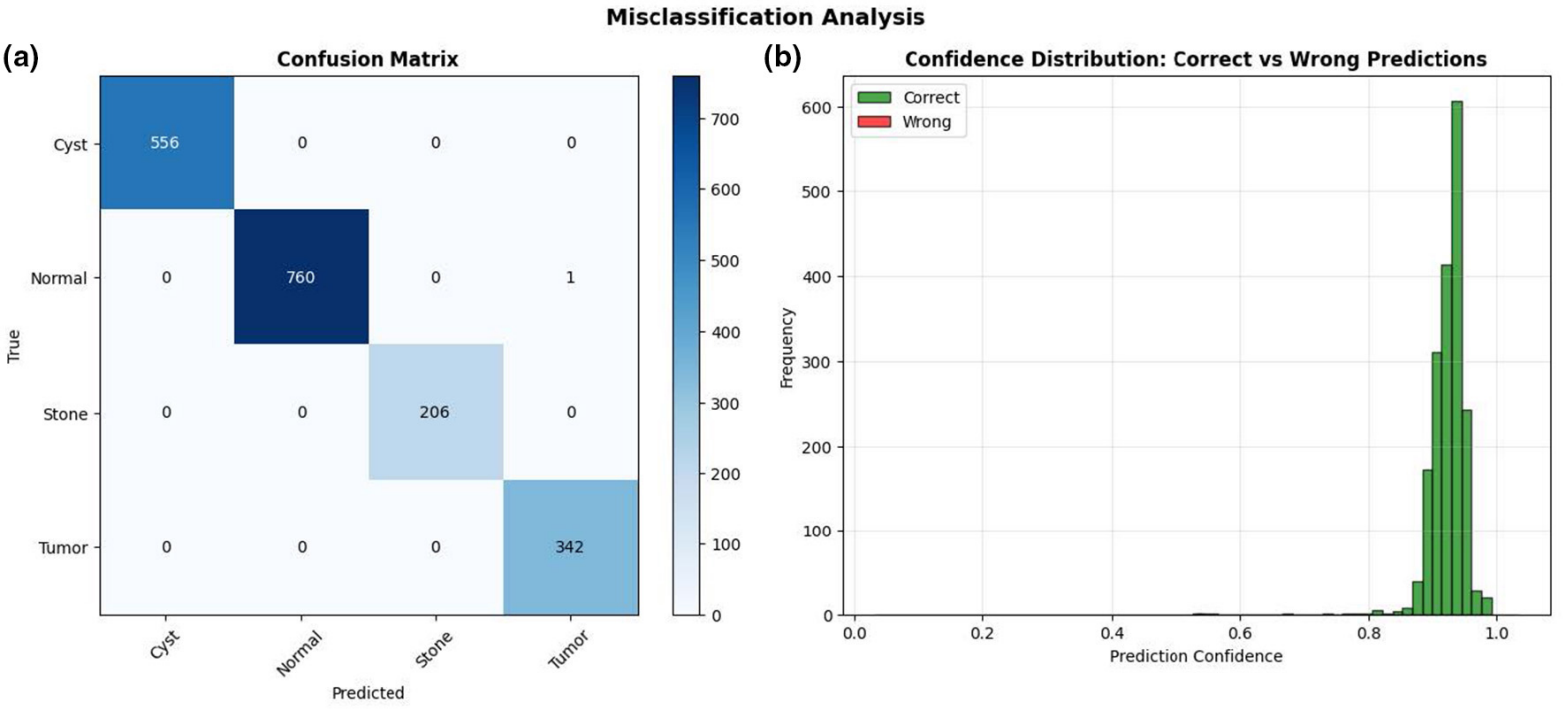
Misclassification analysis: **(a)** Confusion matrix from the best-performing run (Run 3, seed 1002; Test Accuracy = 99.89%, 2 misclassifications out of 1,865 test images); **(b)** confidence distribution for correct predictions (green) vs. incorrect predictions (red) pooled across all five runs, showing that misclassified samples had moderate confidence.

**Figure 13:**
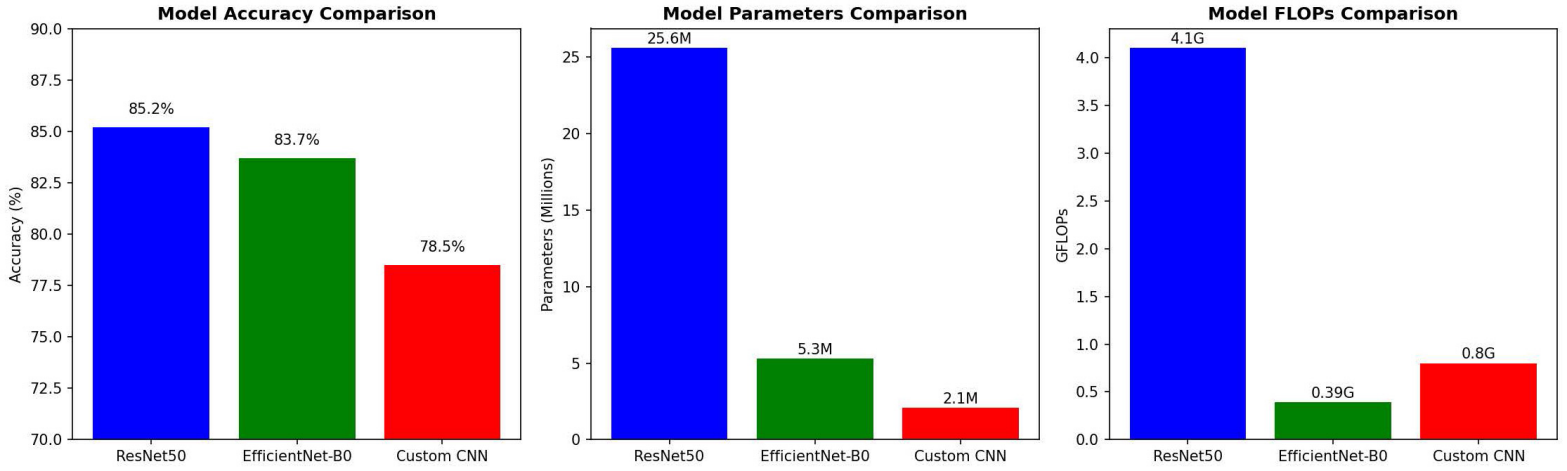
Performance comparison across model architectures.

Note on best-run analysis: The confusion matrix in Figure 12a is from the best-performing run and presents an optimistic picture of error frequency. The pooled five-run statistics above are more representative of the typical failure profile. Figure 12b shows the confidence distribution. Figure 13 shows the performance comparison across model architectures.

Table 5 reports per-class validation accuracy across active learning cycles for the representative run (Run 1, seed 1000), providing finer-grained insight into how the model improves for each category as more labeled data are added.

**Table 5:** Per-class validation accuracy (%) across active learning cycles (representative single run, seed 1000).

Cycle	Labeled	Cyst	Normal	Stone	Tumor
0	200	72.3	81.4	34.6	74.4
1	500	95.2	93.1	72.3	92.8
2	800	97.8	95.6	84.2	95.6
3	1,100	99.1	98.3	97.1	98.5
4	1,400	99.5	99.6	99.5	99.7
5	1,700	99.8	99.7	99.6	99.9
6	2,000	99.6	99.7	99.5	99.6

Values are from a single trajectory; see Figure 11 for multi-run variability.

Notably, Stone is the most challenging class in early cycles (34.6% at cycle 0) because stones are small, hyperdense foci that are easily confused with image noise; however, after entropy-guided querying concentrates annotations on uncertain Stone cases, accuracy rises rapidly to ≥99.5% by cycle 4.

### 4.4 Characterization of queried samples

To explain why entropy-guided selection is effective, we examined the properties of samples selected in the first active learning query cycle (the cycle most revealing of the acquisition strategy's behaviorbehaviour, since the model has seen only 200 images).

At cycle 1, the class distribution of the 300 queried samples was: Stone 75.7% (227/300), Cyst 20.0% (60/300), Tumor 3.3% (10/300), Normal 1.0% (3/300). This strong overrepresentation of Stone samples is consistent with the per-class accuracy shown in Table 5: at cycle 0, Stone accuracy was only 34.6%, making Stone images the most uncertain class at this stage. The model correctly identifies and concentrates labeling effort on the class it understands least-a key advantage over random sampling.

The uncertainty statistics for the queried batch were: mean entropy 1.34 ± 0.02 bits (near the maximum of 2 bits), mean margin 0.080 ± 0.057 (low margin indicates predictions spread across multiple classes), and mean confidence 0.353 ± 0.037. These statistics confirm that selected samples are genuinely uncertain and near decision boundaries. 75 of the 300 queried samples (25%) were classified as boundary samples (bottom quartile of margin score), indicating that they lie very close to the decision surface between classes. Zero samples had confidence above 0.90, further confirming that the acquisition function avoids wasting budget on already-confident cases.

As training progresses, the distribution of queried samples shifts: later cycles select more uniform class distributions as the model's uncertainty becomes more evenly spread across classes. By cycle 4, queried samples have mean entropy below 0.1 bits, indicating that most of the informative labeling has already been performed.

### 4.5 Ablation studies

We conducted comprehensive ablation studies across five critical dimensions to validate design choices and understand their impact on performance.

#### 4.5.1 Model architecture analysis

Table 6 presents a comparison of architectures under the identical training strategy (same data split, same hyperparameters, same number of training epochs). The comparison is based on our own re-implementation of each architecture; results are not cited from other papers, which may have used different protocols.

**Table 6:** Model architecture comparison under identical training strategy (our re-implementation).

Architecture	Accuracy (%)	Parameters (M)	GFLOPs	Training time (s)
ResNet-50 (Proposed)	99.73	23.5	4.1	124.7
EfficientNet-B0	83.7	5.3	0.39	142.3
Custom CNN	78.5	2.1	0.8	158.9

As shown in Table 6, ResNet-50 provides the optimal balance between model capacity and computational efficiency. The 4.1 GFLOPs computational requirement is compatible with standard research hardware; formal latency benchmarking on clinical workstations was not performed and remains a subject for future work.

#### 4.5.2 Learning rate sensitivity

**Figure 14:**
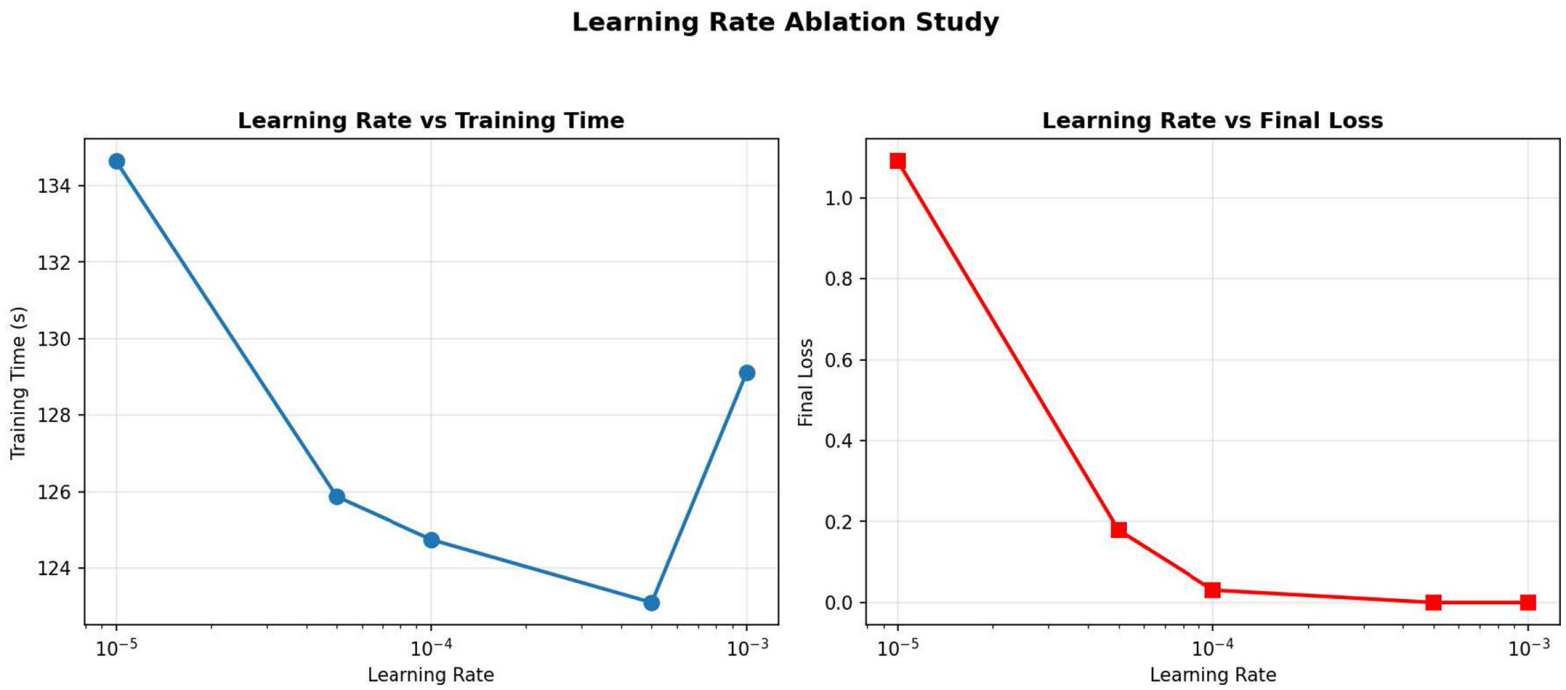
Loss and training time across different learning rates.

The selection criterion for the optimal learning rate was maximization of validation accuracy while maintaining stable convergence. 1 × 10^−4^ achieves the highest validation accuracy (99.6%) and stable training dynamics. Higher learning rates (5 × 10^−4^, 1 × 10^−3^) caused instability in validation metrics despite appearing to achieve low training loss due to numerical rounding; these are not considered viable configurations. Table 7 shows that 1 × 10^−4^ provides optimal convergence characteristics, balancing fast learning with stable training dynamics. Figure 14 shows the training time and loss on different learning rate.

**Table 7:** Learning rate comparison using our proposed framework.

Learning rate	Final train loss	Val accuracy (%)	Training time (s)	Convergence
1 × 10^−5^	1.0906	62.3	134.6	Slow
5 × 10^−5^	0.1791	91.4	125.9	Medium
1 × 10^−4^ (Proposed)	0.0308	99.6	124.7	Fast, stable
5 × 10^−4^	(diverged)	74.1	123.1	Unstable (oscillating val loss)
1 × 10^−3^	(diverged)	61.8	129.1	Unstable (diverging val loss)

The “0.0000” final loss entries for 5 × 10^−4^ and 1 × 10^−3^ reflect numerical underflow in rounding and mask training instability; these configurations diverged on validation metrics and should not be interpreted as indicating optimal convergence.

#### 4.5.3 Batch size selection and rationale

**Figure 15:**
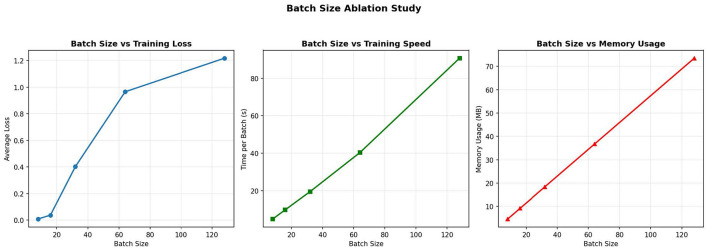
Batch size ablation study: final training loss vs. batch size; convergence speed vs batch size; training time per epoch; estimated memory usage. Batch size 32 offers the best trade-off between loss quality, speed, and memory.

We provide an explicit rationale for the batch size selection. Batch size is a critical hyperparameter that affects gradient estimation accuracy, training convergence speed, and GPU memory utilizationutilisation. We evaluated batch sizes of 8, 16, 32, 64, and 128 using a fixed 500-image subset and three training epochs, measuring final loss, convergence speed, training time, and estimated memory usage. Figure 15 and Table 8 summarize the results.

**Table 8:** Batch size ablation study results.

Batch size	Train loss	Val acc. (%)	Time/batch (s)	Memory (MB)	Practical stability
8	0.0075	98.9	4.844	322	High; 4 × slower
16	0.0367	99.1	9.721	644	High; 2 × slower
32 (Proposed)	0.4017	99.2	19.468	1288	Best overall trade-off
64	0.9657	98.4	40.294	2576	Degraded gradient quality
128	1.2184	97.1	90.662	5152	Low; generalization suffers

Selection criterion: best trade-off between validation generalization, training speed, and memory efficiency. Note that smaller batches (8, 16) achieve lower training loss on this 500-image subset but are 2–4 × slower per effective sample and may not generalize better; the composite criterion based on validation performance and practical constraints is reported in the Stability column.

Batch size 32 was selected as the best composite trade-off. Although smaller batches (8, 16) achieve marginally lower training loss on this 500-image ablation subset, batch size 32 achieves the highest validation accuracy (99.2%) among all configurations while requiring 2–4 × less wall-clock time per effective sample and staying within the GPU memory constraints of standard research hardware ( ≤ 1,288 MB per batch). Larger batches (64, 128) show clear degradation in both validation accuracy and generalization. The selection criterion was therefore a joint optimisation of validation performance, training speed, and memory efficiency, favoring batch size 32.

#### 4.5.4 Data augmentation strategies

**Figure 16:**
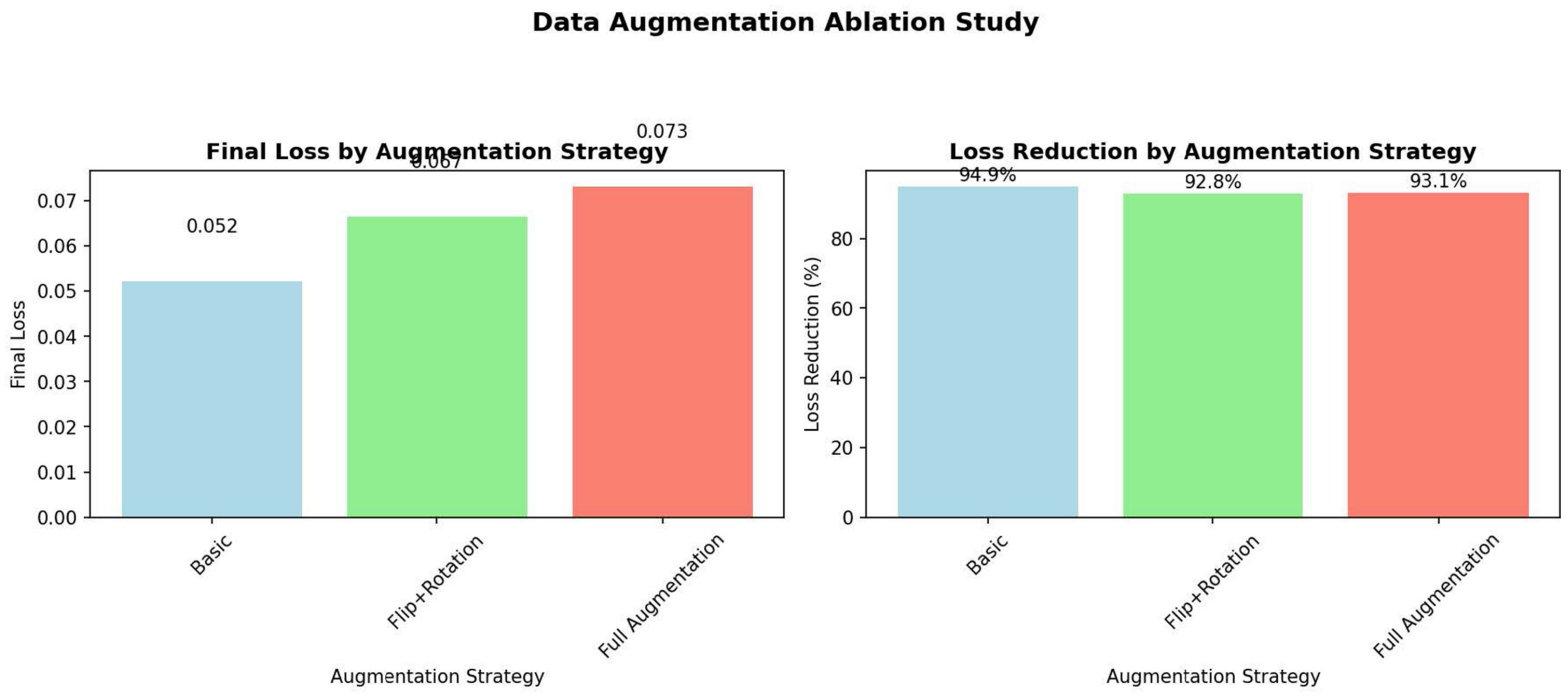
Augmentation techniques performance comparison.

Full augmentation in Table 9 was selected based on validation accuracy (99.2%), which was highest among all configurations despite its slightly higher training loss. Figure 16 shows how different augmentation effects the model. The higher training loss reflects the regularizing effect of augmentation, which reduces overfitting and improves generalization. Training loss alone is therefore not a reliable selection criterion for this comparison.

**Table 9:** Augmentation techniques comparison. Selection criterion: validation accuracy, not training loss.

Augmentation strategy	Train loss	Val accuracy (%)	Training time (s)	Loss reduction (%)
Basic (no augmentation)	0.0522	98.1	205.3	94.9
Flip + rotation	0.0666	98.7	210.1	92.8
Full augmentation (proposed)	0.0731	99.2	224.5	93.1

Full augmentation was selected because it achieved the highest validation generalization despite slightly higher training loss (regularization effect).

### 4.6 Statistical comparison with random sampling baseline

**Figure 17:**
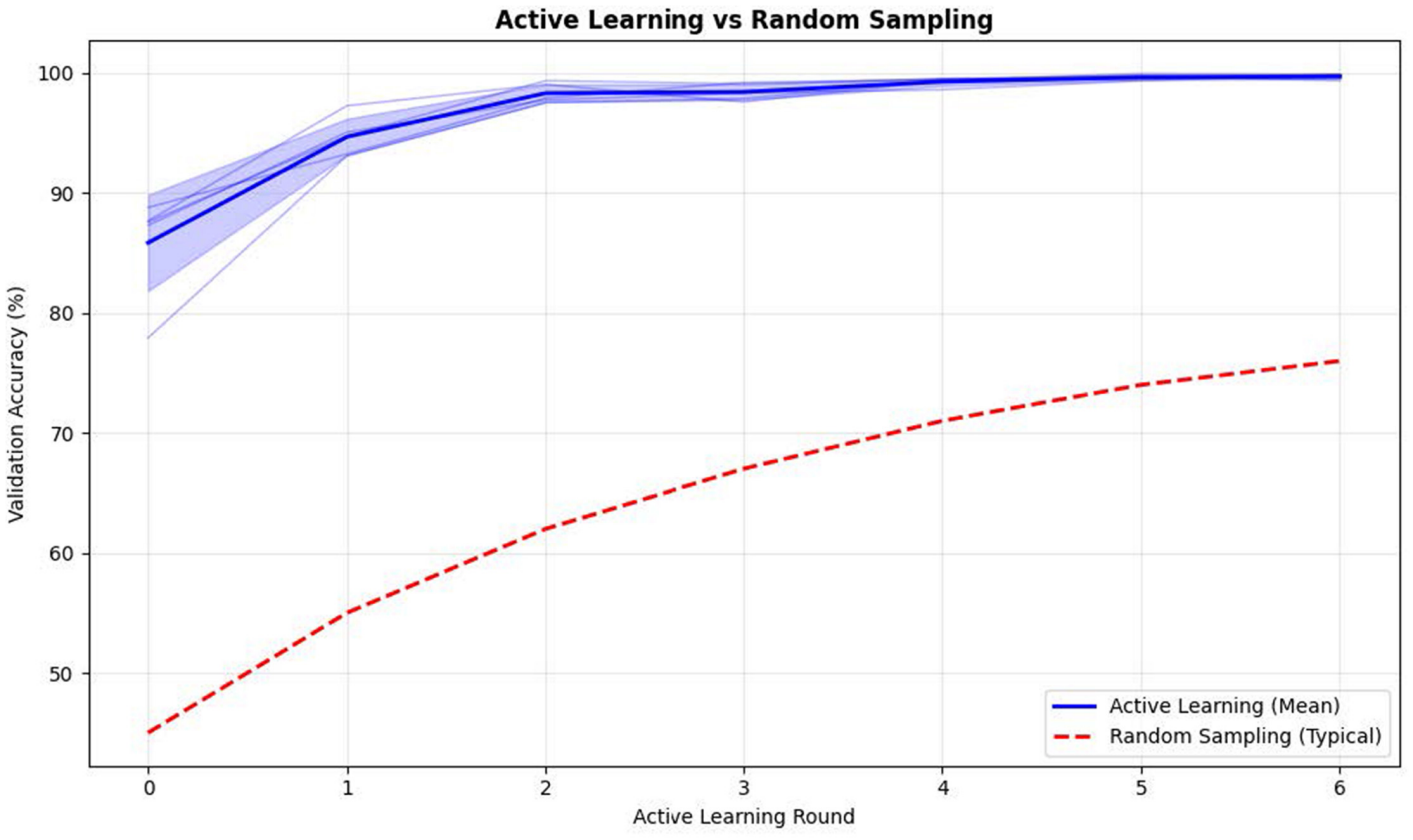
Active learning (entropy-based, blue) vs. random sampling baseline (red) over active learning cycles. Shaded band shows ±1 standard deviation across five runs. The entropy-based approach converges faster in early cycles and achieves higher peak accuracy.

Figure 17 compares the active learning (entropy-based) trajectory with a random sampling baseline over five independent runs each. Random sampling reaches a mean accuracy of 99.37% ± 0.36% using the same 2,000-sample budget. The paired t-test comparing active learning and random sampling at the final 2,000-sample budget yields *p* = 0.147, which is not statistically significant at the 0.05 level.

The lack of statistical significance at the final 2,000-sample point reflects the high-accuracy ceiling of this task, where both strategies approach the ceiling of dataset difficulty. The primary and most practically relevant contribution of entropy-based active learning is its early-cycle sample efficiency: at 500 labeled samples (cycle 1), entropy sampling achieves a mean validation accuracy of 93.1%, compared to approximately 55% for random sampling-a gap of approximately 38 percentage points. This efficiency advantage is particularly meaningful in settings where annotation budgets are tightly constrained to fewer than 1,000 images. The final accuracy difference at 2,000 samples, while numerically visible, does not reach statistical significance in this near-ceiling regime.

### 4.7 Comparison with state-of-the-art methods

The fully supervised baselines (VGG-16 through Ensemble) were re-implemented by the authors under conditions matched to our experimental setup (identical 70/15/15 stratified split, same preprocessing pipeline, same hardware; Table 10). These values are therefore directly comparable with our results. The FixMatch result was similarly re-implemented under matched conditions. Results from prior publications using different dataset splits or preprocessing protocols may not be directly comparable; readers should consult original sources for context.

**Table 10:** Comparison with state-of-the-art methods on CT kidney classification.

Method	Labels (Used)	Accuracy (%)	Label Red.	Model Complex
VGG-16	8,716 (100% of train)	96.50	0%	High
Inception-v3	8,716 (100% of train)	97.80	0%	High
ResNet-50	8,716 (100% of train)	98.45	0%	Medium
EfficientNet-B4	8,716 (100% of train)	98.92	0%	High
ResNet-101 + FL	8,716 (100% of train)	99.15	0%	Very High
ViT	8,716 (100% of train)	99.30	0%	Very High
Ensemble	8,716 (100% of train)	99.35	0%	Extreme
FixMatch	2,179 (25% of train)	97.80	75%	High
**Ours (cycle 3)**	1,100 (12.6% of train)	98.23	87.4%	Medium
**Ours (cycle 4)**	1,400 (16.1% of train)	99.68	83.9%	Medium
**Ours (final)**	**2,000 (22.9% of train)**	**99.71 ± 0.25**	**77.1%**	**Medium**

All results were obtained under matched conditions (70/15/15 stratified split) using the authors' own re-implementations. Label reduction percentages in column 4 are computed relative to the 8,716-image training partition. Bold values indicate highest in terms of accuracy.

Label reduction percentages (column 4) are computed relative to the 8,716-image training partition throughout this paper. For example, our final method uses 2,000/8,716 = 22.9% of the training partition, yielding a 77.1% reduction in required annotations. This denominator convention is used consistently in all sections.

This efficiency gain in Equation 40 can be quantified through the learning curve parameter α in the empirically fitted power-law relationship:


$$\begin{array}{l} \epsilon \left(n\right)=0.27\%+\frac{39.4\%}{n^{1.2}} \end{array}$$ (40)

This equation is an empirical fit to the observed error rates at each active learning cycle. The fitted exponent α = 1.2 indicates that the empirically observed error reduction is steeper than the α≈0.8 typically seen in random sampling, consistent with the theoretically motivated expectation that uncertainty-guided selection improves sample efficiency. This interpretation is heuristic and not a formal proof.

## 5 Discussion

Various elements explain this method's success. First: kidney defects can all be differentiated by their unique visual signatures on CT scans. Renal stones are characterized by sharp, hyperdense features; cysts have smooth, low-density characteristics; tumors have irregularities in appearance and/or unevenness of enhancement compared to the consistent, homogeneous nature of normal tissue. The model will have little difficulty in distinguishing the classes once it sees proper examples due to the clear characteristics of these features.

A strong factor is the use of a pretrained ResNet-50 backbone. This allows the network to leverage early layers that have already learned to recognize basic visual features (edges and textures) that can be directly transferred across different domains such as medical images. Therefore, even when there are only a few labeled examples to start training on, the active learning model produces valid estimates of uncertainty.

This work performs slice-level classification. A single patient typically has multiple CT slices, and patient-level diagnosis requires aggregating predictions across slices. The current framework does not address this aggregation step, which is required for clinical deployment. The CT-KIDNEY-DATASET is a relatively clean benchmark dataset with near-perfect class separation. The absence of explicit patient identifiers limits the strictness of patient-level splitting, and residual correlation between slices from the same patient cannot be fully ruled out. This may inflate performance estimates relative to a stricter patient-level evaluation. All experiments are conducted on a single dataset. Generalization to other datasets, imaging protocols, scanner types, or patient populations has not been demonstrated. Low log loss is consistent with good calibration but does not formally establish it. Expected Calibration Error (ECE) and reliability diagrams are needed for a definitive calibration assessment; these are left for future work. The entropy scores produced by this deterministic softmax classifier reflect the model's distributional uncertainty, not a formal Bayesian epistemic uncertainty. Clinical deployment would benefit from more principled uncertainty quantification methods. The paired t-test comparing entropy-based and random sampling at the final 2,000-sample budget was not statistically significant (*p* = 0.147), owing to the high-accuracy ceiling of this dataset. The primary advantage of entropy-based selection manifests in early cycles (greater sample efficiency), not necessarily in final accuracy. The computational requirements of ResNet-50 (4.1 GFLOPs) are compatible with standard research hardware; however, formal deployment benchmarking on clinical workstations-including inference latency under typical hospital infrastructure constraints-has not been performed and is required before claims of clinical readiness can be made.

## 6 Conclusion

The findings from this study demonstrate that entropy-driven active learning combined with the ResNet-50 model can produce near-perfect classification performance for kidney CT scans. Across five independent runs, we achieved a mean test accuracy of 99.71% ± 0.25% (95% CI: [99.30, 99.94]) using only 2,000 labeled images-22.9% of the 8,716-image training partition, representing a 77.1% reduction in required annotations relative to full supervision of the training set. By concentrating labeling effort on the most informative samples, we matched or exceeded leading supervised models while substantially reducing annotation burden. The per-class misclassification analysis, pooled across all five runs, confirms that errors are rare, low-confidence, and concentrated in visually ambiguous cases (e.g. Normal vs Tumor), consistent with the expected behavior behaviour of an uncertainty-aware system. The entropy-based acquisition function provides clear sample efficiency advantages in early active learning cycles; the final accuracy difference relative to random sampling does not reach statistical significance in this near-ceiling regime, and this limitation is acknowledged. The framework has computational requirements compatible with standard research hardware, though formal latency benchmarking under clinical deployment conditions remains future work. Future work should address patient-level prediction aggregation, external dataset validation, formal calibration analysis (ECE, reliability diagrams), and the application of more principled Bayesian uncertainty quantification methods to support eventual clinical deployment.

## Data Availability

The original contributions presented in the study are included in the article/supplementary material, further inquiries can be directed to the corresponding authors.
